# Magnetic-resonance-based measurement of electromagnetic fields and conductivity *in vivo* using single current administration—A machine learning approach

**DOI:** 10.1371/journal.pone.0254690

**Published:** 2021-07-22

**Authors:** Saurav Z. K. Sajib, Munish Chauhan, Oh In Kwon, Rosalind J. Sadleir

**Affiliations:** 1 School of Biological Health System Engineering, Arizona State University, Tempe, Arizona, United States of America; 2 Department of Mathmatics, Konkuk University, Seoul, Korea; Zhejiang University, CHINA

## Abstract

Diffusion tensor magnetic resonance electrical impedance tomography (DT-MREIT) is a newly developed technique that combines MR-based measurements of magnetic flux density with diffusion tensor MRI (DT-MRI) data to reconstruct electrical conductivity tensor distributions. DT-MREIT techniques normally require injection of two independent current patterns for unique reconstruction of conductivity characteristics. In this paper, we demonstrate an algorithm that can be used to reconstruct the position dependent scale factor relating conductivity and diffusion tensors, using flux density data measured from only one current injection. We demonstrate how these images can also be used to reconstruct electric field and current density distributions. Reconstructions were performed using a mimetic algorithm and simulations of magnetic flux density from complementary electrode montages, combined with a small-scale machine learning approach. In a biological tissue phantom, we found that the method reduced relative errors between single-current and two-current DT-MREIT results to around 10%. For *in vivo* human experimental data the error was about 15%. These results suggest that incorporation of machine learning may make it easier to recover electrical conductivity tensors and electric field images during neuromodulation therapy without the need for multiple current administrations.

## Introduction

Magnetic resonance based methods have recently been used to image parameters characterizing low-frequency electrical current flow in the human body, including current density and conductivity tensors [1]. These methods proceed from the observation that magnetic flux density components caused by externally applied current flow can be recovered from MR phase images [2, 3]. Because only one component of magnetic flux density, *B*_*z*_, the component along the longitudinal axis of an MRI system, can be measured conveniently, specialized techniques have been developed to recover conductivity information from these data [1]. These magnetic resonance electrical impedance tomography (MREIT) approaches have included *J*-substitution [4], sensitivity-based methods [5], and the Harmonic *B*_*z*_ method [6, 7]. More recently, additional information provided from diffusion tensor (DT) images have been combined with *B*_*z*_ measurements to allow *in vivo* imaging of conductivity tensors in the human brain [8]. This DT-MREIT technique involves reconstruction of a position-dependent scale factor that is then multiplied with diffusion tensor images to produce conductivity tensor information [9, 10].

The aim of the present study was to develop a data processing method to enable quantitative visualization of electromagnetic field and property distribution during transcranial electrical current stimulation (tES) therapy. To generate therapeutic electromagnetic fields, a stimulation current of around 1–2 mA is injected via pairs of surface electrodes located over target brain structures [11, 12]. Electromagnetic models of the human body are of great use in planning tES treatments, and MREIT methods provide a means of directly measuring tES current paths. Measurement of accurate, subject-specific conductivity volumes could increase model specificity and therefore planning efficacy. The Harmonic *B*_*z*_ method and DT-MREIT typically require two independent current administrations to reconstruct unique conductivity distributions. However, in many situations, including experimental tES, or in deep brain stimulation (DBS), it is not usual or often possible to use two current administrations. Therefore, it is of great interest to establish methods that can work within existing stimulation frameworks.

Previous MREIT studies reconstructing tES current density or conductivity distributions in human subjects have used two current pairs that were located approximately in a single transverse plane [8, 13]. This is advantageous for MREIT since the majority of the current flow information is contained in *B*_*z*_ data (i.e. *J*_*z*_ is small). Most tES montages do not use electrodes in a single plane. Further, depending on where the electrodes are located on the head, current flow may still involve a non-negligible *J*_*z*_ component. An example of this montage is the F3-F4 montage that has been used to test cognitive and memory tasks [14]. While the electrodes are nominally in the same plane of the head, its shape results in a *J*_*z*_ component amplitudes similar to either *J*_*x*_ or *J*_*y*_. Accuracy of existing techniques depends directly on the relative magnitude of *J*_*z*_ [15, 16]. There is therefore a need to develop alternative and more flexible methods for leveraging the availability of *B*_*z*_ images where there may be only data from a single or multiple electrode pairs involving significant *J*_*z*_. This study therefore represents a first step towards tackling the problem of how current density and conductivity distributions may be obtained using incomplete data.

Kwon *et al*. [17] proposed an iterative method for obtaining tES current density distributions by minimizing the difference between current densities estimated from the measured *z*-component of the magnetic flux density with model-predicted current densities. However, it was not possible to obtain the reconstructed ‘apparent’ conductivity tensor and electric field distribution for tES currents in [17], mainly due to the lack of stable methods for reconstructing the DT-MREIT scale factor from a single current injection. The problem of conductivity reconstruction using single-current-administration *B*_*z*_ data is generally ill-posed [1]. Fortunately, it has been proven in [15] it is possible to reconstruct the internal conductivity distribution (and also the scale-factor distribution in DT-MREIT) uniquely from single-current *B*_*z*_ data if the conductivity at the boundary surface is known. Lee *et al*. [18] proposed an iterative method that extended the original diffusion weighted (DW) *J*-substitution algorithm [19] to image electrical conductivity distributions during tES. However, this method depended on the initial choice of scale factor value to ensure its convergence [20].

Machine learning methods are presently being adopted for nonlinear ill-posed inverse problems in medical imaging such as reconstruction of MR images from partial data [21, 22], electrical impedance tomography [23–25] and magnetic resonance electrical properties tomography [26, 27], either for direct reconstruction from raw data, or in method postprocessing, mainly because of its ability to approximate non-linear functions from finite input-output data sets. However, these approaches have not yet been explored in DT-MREIT reconstruction.

In transcranial electrical stimulation, montages do not generally involve administration of multiple linearly independent currents, and most often use only one pair of large electrodes located over target brain structures [12]. Generally, tES protocols involve alternating or direct current applied with amplitudes of up to 4 mA [28]. The use of neuromodulation therapies also provides a unique opportunity to image conductivity distributions, which may be of use in constructing subject-specific computational models used for treatment planning, whereas conventional models have used conductivities for brain tissues derived from literature values [29].

In this work, we extend tES-based approaches to finding electromagnetic parameters, using *B*_*z*_ data to demonstrate an ability to reconstruct conductivity tensors from only one current injection. In addition to using *a*Â *priori* information from diffusion tensor imaging, we used elementary machine learning, simulations of additional data obtained from computational models, and mimetic algorithm approaches to obtain estimates of conductivity, electric field and current density distributions. We compare these single-current results to those obtained using standard two-current reconstructions. Feasibility of the method is demonstrated using phantom and *in vivo* human results and in-plane electrode locations. It is anticipated that extension of the methods employed here may be useful in measuring electric fields and conductivity distributions during tES therapy using arbitrary electrode locations.

## Theory

Key theory involved in machine-learning-aided DT-MREIT reconstructions performed in this paper is described in the sections below. The assumed relation between diffusion tensor and conductivity tensors is shown, followed by a description of how the scaling factor may be reconstructed using a mimetic method based on the Kirchhoff voltage law. Finally, training and correction of reconstructions using an artificial neural network is outlined.

### Relation between water diffusion and conductivity tensors

The effective water diffusion tensor **D** measured within a voxel using pulsed-gradient-spin echo sequences [30] can be written as a 3 × 3 positive definite symmetric matrix:

$$\begin{array}{c} D=S_{D}\Lambda_{D}S_{D}^{T}\;\text{with}\;\Lambda_{D}=(\begin{array}{ccc} \lambda_{D,1} & 0 & 0\\ 0 & \lambda_{D,2} & 0\\ 0 & 0 & \lambda_{D,3} \end{array}) \end{array}$$ (1)

where the column vectors forming **S**_*D*_ = {**s_1_**, **s_2_**, **s_3_**} are the eigenvectors of **D**, the superscript *T* denotes transpose and λ_*D*,*k*_ for *k* = 1,2,3 are the corresponding eigenvalues. There is no known relationship between water diffusion and electrical conductivity in a free electrolyte solution. However, since the water, ions and other charged particles coexist in the same microscopic environment, Tuch *et al.* [31] suggested the eigenvalues λ_*C*,*k*_ at low frequency (<1 kHz) conductivity tensor **C** were related to diffusion coefficients as

$$\begin{array}{c} \begin{array}{c} \lambda_{C,k}=\frac{C_{e}}{d_{e}}[\lambda_{D,k}(\frac{d_{i}}{3d_{e}}+1)+\lambda_{D,k}^{2}\frac{d_{i}}{3d_{e}^{2}}-\frac{2}{3}d_{i}]+O\left(d_{i}^{2}\right).\;\;k=1,2,3 \end{array} \end{array}$$ (2)

Here, **C** *_e_* is the extra-cellular conductivity, *d*_*i*_ and *d*_*e*_ are the intra- and extracellular diffusion coefficients, respectively, and $O(d_{i}^{2})$ is bounded as $d_{i}^{2}$ tends to infinity. By neglecting intracellular diffusion in relation (2), as in [8–10, 19, 32, 33] the effective conductivity tensor may be expressed as

$$\begin{array}{c} C=S_{D}\;\Lambda_{C}S_{D}^{T}=\eta D\;\text{with}\;\Lambda_{C}=(\begin{array}{ccc} \lambda_{C,1} & 0 & 0\\ 0 & \lambda_{C,2} & 0\\ 0 & 0 & \lambda_{C,3} \end{array}) \end{array}$$ (3)

where the position dependent scale factor *η* can be determined by measuring magnetic flux density induced due to an externally injected current [9, 10].

### Reconstruction of scale factor *η* using discretization of Faraday’s law

We represent the imaged domain Ω as a stack of axial slices Ω_*t*_ arranged perpendicular to the *z*-axis such that $\Omega ={⋃}_{t\in (-H_{0},H_{0})}\Omega_{t}\;\text{where}\;\Omega_{t}=\{\Omega ⋂\left\{\left(x,y,z\right)\in R^{3}{|}_{z=t}\right\}$ and the entire slice package covers a region of total thickness 2*H*_0_. The divergence-free property of electrical current density, **J** = −**C**∇*u* = −*η***D**∇*u*, combined with an externally-injected current density *g* applied to the domain boundary leads to the following elliptic partial differential equation

$$\begin{array}{c} \left\{ \begin{array}{c} \nabla \cdot J=\nabla \cdot (-\eta D\nabla u)=0\;\;\text{in}\;\Omega \\ J\cdot n=-\eta D\nabla u\cdot n=g\;\;\;\;\;\text{on}\;\partial \Omega \end{array}\right. \end{array}$$ (4)

where *u* is a voltage distribution, and **n** = (*n*_*x*_, *n*_*y*_, *n*_*z*_) is the outward-normal unit vector defined at the boundary ∂Ω of the domain Ω.

**Fig 1:**
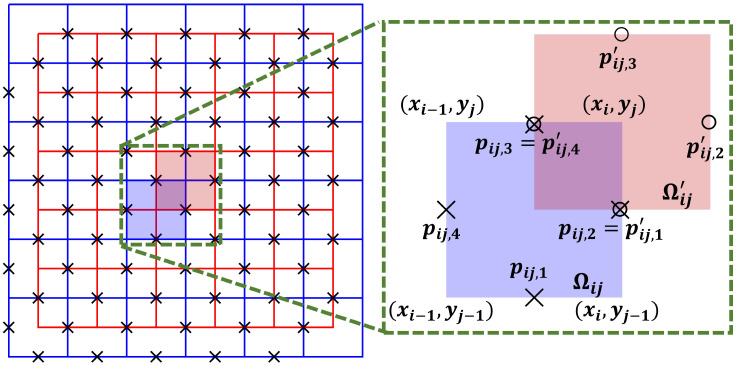
Schematic of the dual-loop network. The primary loop is shaded in blue and the secondary (primed) loop is shaded in red.

The Kirchhoff voltage law (KVL) is a low-frequency corollary of Faraday’s law which states that sum of the voltage drops or electric field components around a closed path is zero. In this work, we used the KVL to reconstruct scale factors, *η*, within each slice of a three-dimensional domain using estimated current densities and diffusion tensor data. To use the KVL we discretized each slice Ω_*t*_ as a rectangular grid $\Omega_{t}={⋃}_{i=1,j=1}^{N_{x},N_{y}}\Omega_{ij}$ (Fig 1). Since **J** = −**C**∇u = −*η***D**∇*u*, the quantity $\frac{D^{-1}J}{\eta }$ has the dimensions of the electric field, and via the KVL we have that

$$\begin{array}{c} \begin{array}{c} \frac{{\left(D^{-1}J\right)}_{x}\left(p_{ij,1}\right)}{\eta (p_{ij,1})}+\frac{{\left(D^{-1}J\right)}_{y}\left(p_{ij,2}\right)}{\eta (p_{ij,2})}-\frac{{\left(D^{-1}J\right)}_{x}\left(p_{ij,3}\right)}{\eta (p_{ij,3})}-\frac{{\left(D^{-1}J\right)}_{y}\left(p_{ij,4}\right)}{\eta (p_{ij,4})}=0 \end{array} \end{array}$$ (5)

where *p*_*ij*,1_, *p*_*ij*,2_, *p*_*ij*,3_, *and* *p*_*ij*,4_ are points at the center of the closed loop defining the rectangular region Ω_*ij*_ located at coordinates (*x*_*i*−1_, *y*_*j*−1_), (*x*_*i*_, *y*_*j*−1_), (*x*_*i*_, *y*_*j*_), (*x*_*i*−1_, *y*_*j*_) respectively, as shown in Fig 1. It is also possible to design another loop, $\Omega_{ij}^{{}^{\prime }}$, such that

$$\begin{array}{c} \begin{array}{c} \frac{{\left(D^{-1}J\right)}_{x}\left(p_{ij,1}^{{}^{\prime }}\right)}{\eta (p_{ij,1}^{{}^{\prime }})}+\frac{{\left(D^{-1}J\right)}_{y}\left(p_{ij,2}^{{}^{\prime }}\right)}{\eta (p_{ij,2}^{{}^{\prime }})}-\frac{{\left(D^{-1}J\right)}_{x}\left(p_{ij,3}^{{}^{\prime }}\right)}{\eta (p_{ij,3}^{{}^{\prime }})}-\frac{{\left(D^{-1}J\right)}_{y}\left(p_{ij,4}^{{}^{\prime }}\right)}{\eta (p_{ij,4}^{{}^{\prime }})}=0 \end{array} \end{array}$$ (6)

where $p_{ij,1}^{{}^{\prime }}=(x_{i},\frac{y_{j-1}+y_{j}}{2})$, $p_{ij,2}^{{}^{\prime }}=(\frac{x_{i}+x_{i+1}}{2},y_{j})$ $p_{ij,3}^{{}^{\prime }}=(x_{i},\frac{y_{j}+y_{j+1}}{2})$, and $p_{ij,4}^{{}^{\prime }}=(\frac{x_{i-1}+x_{i}}{2},y_{j})$ are the loop vertices (Fig 1).

Note that the dual loop network is designed such a way that *x*, *y*-components of (**D**^−1^ **J**) vectors at the points $p_{ij,1}^{{}^{\prime }},\;\text{and}\;p_{ij,2}$ are used simultaneously to determine *η* values at that position [34].

The representative scale factor in overlapping loops can than be found by combining the identities (5) and (6) to form a dual-loop network i.e.

$$\begin{array}{c} \begin{array}{c} \frac{{\left(D^{-1}J\right)}_{x}\left(p_{ij,1}\right)}{\eta (x_{i},y_{j-1})}+\frac{{\left(D^{-1}J\right)}_{y}\left(p_{ij,2}\right)}{\eta (x_{i},y_{j})}-\frac{{\left(D^{-1}J\right)}_{x}\left(p_{ij,3}\right)}{\eta (x_{i},y_{j})}-\frac{{\left(D^{-1}J\right)}_{y}\left(p_{ij,4}\right)}{\eta (x_{i-1},y_{j})}=0 \end{array} \end{array}$$ (7a)

$$\begin{array}{c} \begin{array}{c} \frac{{\left(D^{-1}J\right)}_{x}\left(p_{ij,1}^{{}^{\prime }}\right)}{\eta (x_{i},y_{j})}+\frac{{\left(D^{-1}J\right)}_{y}\left(p_{ij,2}^{{}^{\prime }}\right)}{\eta (x_{i+1},y_{j})}-\frac{{\left(D^{-1}J\right)}_{x}\left(p_{ij,3}^{{}^{\prime }}\right)}{\eta (x_{i},y_{j+1})}-\frac{{\left(D^{-1}J\right)}_{y}\left(p_{ij,4}^{{}^{\prime }}\right)}{\eta (x_{i},y_{j})}=0 \end{array} \end{array}$$ (7b)

With the further assumption that *η* values are known on the boundary, the dual-loop network defines an overdetermined system containing a total of 2(*N*_*x*_ − 2)(*N*_*y*_ − 2) equations over 2(*N*_*x*_ − 2)(*N*_*y*_ − 2) loops or cells and (*N*_*x*_ − 2)(*N*_*y*_ − 2) internal nodes, for each Ω_*t*_.

Using linear interpolation of (**D**^−1^ **J**) vectors at the center of the nodes shown in Fig 1, the right hand side of Eq (8) contains known boundary voltage differences, $B_{p}$ or $B_{s}$ estimated from known boundary *η* and (**D**^−1^ **J**) values around the loop perimeter.

An expression describing the combined primary and secondary loops may be written

$$\begin{array}{c} \left(\begin{array}{c} A_{p}\\ A_{s} \end{array})X=(\begin{array}{c} B_{p}\\ B_{s} \end{array}\right) \end{array}$$ (8)

where the vector X contains inverse scale factor *η* values at each node, and elements of the stiff matrices $A_{p}$ (primary) and $A_{s}$ (secondary) on the left hand side of Eq (8) contain the numerator terms from Eqs (7a) and (7b) respectively.

Solutions for **x** were found using regularization via a least squares method

$$\begin{array}{c} X={\left(A^{T}A+\lambda I\right)}^{-1}A^{T}B. \end{array}$$ (9)

Here, λ represents a regularization parameter, **I** is an identity matrix, and *T* denotes matrix transpose. The matrix $A={\left(A_{p}\;A_{s}\right)}^{T}$ and $B={\left(B_{p}\;B_{s}\right)}^{T}$.

### Correction of dual-loop *η* reconstructions using artificial neural network methods

**Fig 2:**
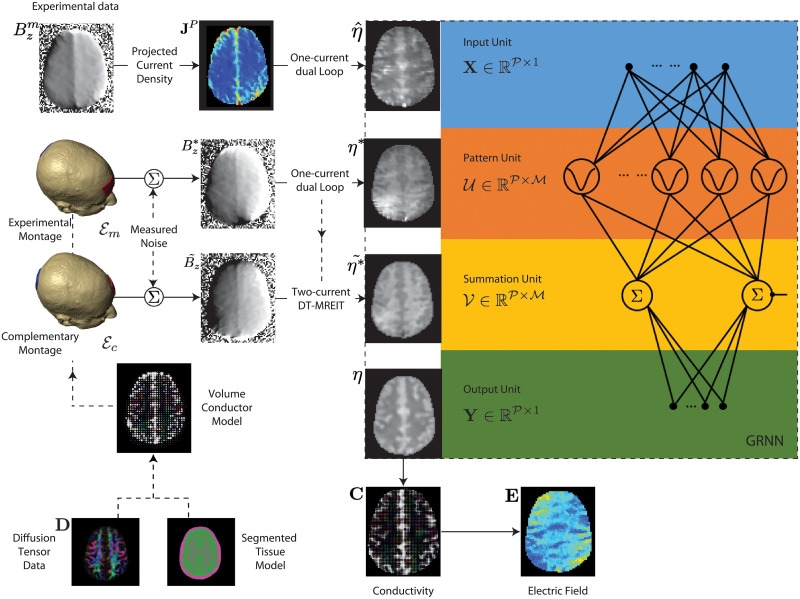
Flow diagram of the proposed method demonstrated for a head-shaped domain.

The reliability of reconstructed *η* values using the dual-loop method with one current injection is limited, due to noise propagating along equipotential lines [34, 35]. Therefore, an artificial neural network (ANN) approach was used to overcome these limitations. ANN methods use a number of sample input-output pairs D={U,V} to create a regressor model of a non-linear artifact function *f* mapping the input to the output. In our application, the function mapped dual-loop output to artifact-free images as

$$\begin{array}{c} f:\{X\in R^{P\times 1}\to Y\in R^{P\times 1}\} \end{array}$$ (10)

where **X** and **Y** are vectorized images of the uncorrected input $\hat{\eta }$ and the corrected output *η*, respectively (Fig 2) and P denotes the number of non-zero voxels in each image. The data set $U\in R^{P\times M}$ in D is the result of M dual-loop-network calculations, each of which is denoted η* or $U_{k},\;k=1,2,3,\ldots M$, and $V\in R^{P\times M}$ consists of M artifact-free *η* images, each denoted ${\tilde{\eta }}^{*}$ or $V_{k}$. Training data are identified using the notation ()*, thus a row of V may also be identified as the image $\tilde{\eta^{*}}$.

We used a generalized regression neural network (GRNN) [36] to construct the functional relationship *f* in (10). The probabilistic GRNN architecture consisted of four layers: an input unit, a pattern unit consisting of Gaussian neurons, a summation unit and an output layer (Fig 2) [36]. For an uncorrected input **X**, the output of the GRNN **Y** (the conditional mean) may be expressed as [36]

$$\begin{array}{c} Y\left(X\right)=\frac{\sum_{k=1}^{M}V_{k}\exp(-\frac{D_{k}^{2}}{2\alpha^{2}})}{\sum_{k=1}^{M}\exp(-\frac{D_{k}^{2}}{2\alpha^{2}})}. \end{array}$$ (11)

In (11), the spread-constant of the Gaussian neurons is denoted by *α*, $V_{k}$ is the *k*-th artifact-free vectorized image ${\tilde{\eta }}^{*}$ and the total number of neurons (samples) in the pattern unit is M [36]. The function $D_{k}$ [36]

$$\begin{array}{c} D_{k}^{2}={\left(X-U_{k}\right)}^{T}(X-U_{k}), \end{array}$$ (12)

measures the distance between the input **X** and the *k*-th training vector, $U_{k}$ k=1,2,3,…M. Eq (11) can be interpreted as relating an expression for the expected output **Y** to a given input data vector X, which is the weighted sum of the artifact-free training data sets $V_{k}$, where for a chosen *α* the weighting-factors are determined by the Euclidian distance between input and the training datasets $U_{k}$ (Eq (12)). Therefore, a particular neuron will produce more weight in the output if it is close to the input data-vector, and vice versa. The spread constant *α* controls the degree of smoothness of the GRNN output.

As noted in [36], the predicted output of the GRNN-neural network in Eq (11) is associated with the underlying joint probability distribution function (pdf). When the distribution function is not known, Specht [36] suggested using a nonparametric estimate of the joint-pdf which depends on the spread constant and the number of samples in the data sets (as well as the number of neurons in the pattern unit). While there is no general rule that predicts the number of samples M required in the pattern unit with a specified degree of accuracy, good prediction results are often obtained with a modest number of samples [37]. Additionally, it has been shown that for a fixed number of data-points P the spread-constant of the joint-pdf gradually decreases as sample size increases [36, 37]. However, with large training data sets, Specht [36] suggests a simple clustering technique to reduce the number of neurons in the pattern unit and avoid unnecessary computational burden in the GRNN network. Cluster centers are estimated by averaging the data samples present in the cluster, which eventually improves the underlying noise characteristics of the center-vector of the Gaussian neurons (Eq 12). In this study all training data sets were used in the pattern unit (Fig 2) to predict *η*.

In the GRNN, the spread-constant *α* is the only parameter which needs to be determined by the training process. In this paper, we determined an optimum *α* by minimizing the mean-squared error (*MSE*) error cost function. For a given *α*, the mean-squared error for the *k*-th sample data, *MSE*_*k*_, is defined as

$$\begin{array}{c} MSE_{k}\left(\alpha \right)=\frac{1}{P}\sum_{j=1}^{P}{\left(V_{k}^{\left(j\right)}-{\hat{Y}}_{k}^{\left(j\right)}\right)}^{2}, \end{array}$$ (13)

where ${\hat{Y}}_{k}$ is the predicted output of the GRNN for the input $U_{k}$.

In [36] an optimum spread-constant, $\hat{\alpha }$ was determined from the training data sets D using a *holdout* method. In the holdout method, for a chosen *α*, a GRNN network is constructed using the data sets, $D\backslash D_{k},k=1,2,3,\ldots M$ and the mean-squared error (13) is calculated by comparing the network prediction using true data $D_{k}=\left\{U_{k},V_{k}\right\}$. This process was then repeated for other samples in the data sets to determine the mean *MSE* value for each *α*. In the final network, the optimum spread-constant $\hat{\alpha }$ value was set to the value producing the smallest *MSE*. In this work, we used the constrained optimization function ‘fminbnd’, implemented in the MatLab optimization toolbox (The MathWorks, Inc., Natick, Massachusetts, United States) to determine the $\hat{\alpha }$ value in the range 10^−3^ < *α* < 10^3^. This value was used in the final network to predict an artifact-free **Y** at a given **X** in (11).

## Methods

Specific techniques used to extract scaling factors from single current applications are described in this section, including assumptions and calculations involved in simulating magnetic flux density data and current density to generate training data and methods used to reconstruct quantities such as current density and electric fields.

### Formation of $B_{z}^{m}$ from MR phase images

In MREIT, the *z*-component of the current induced magnetic flux density *B*_*z*_ is measured using an MRI scanner. The accumulated phase due to the external current injection depends on the induced *B*_*z*_ and current injection duration *T*_*c*_. The complex MR signal density developed using a spin-echo MR pulse sequence can be represented in the spatial domain as

$$\begin{array}{c} S^{\pm }\left(r\right)=\rho \left(r\right)e^{i\delta (r)}e^{\pm i\gamma B_{z}^{m}\left(r\right)T_{c}} \end{array}$$ (14)

where *ρ*(**r**) is the MR magnitude image, *δ*(**r**) represents a systematic phase artifact at position **r**, *γ* is the proton gyromagnetic ratio, and the superscript on *B*_*z*_ represents the experimentally measured data. From Eq (14), the magnetic flux densities generated by positive and negative current injections *I*^±^ can be recovered using

$$\begin{array}{c} B_{z}^{m}\left(r\right)=\frac{1}{2\gamma T_{c}}\text{arg}(\frac{S^{+}\left(r\right)}{S^{-}\left(r\right)}). \end{array}$$ (15)

From the analysis of [3, 38], the noise standard deviation $sd_{B_{z}^{m}}$ in $B_{z}^{m}$ depends on the signal-to-noise ratio (SNR) of the MR magnitude image *Υ*_*ρ*_ and the current injection duration *T*_*c*_ as

$$\begin{array}{c} sd_{B_{z}^{m}}\propto \frac{1}{\gamma T_{c}{ϒ}_{\rho }}. \end{array}$$ (16)

Phantom data for this work were gathered using spin echo MREIT imaging sequences. However, these sequences involve long acquisition times [39] and are inconvenient for human subjects. A faster multi-gradient-echo imaging sequence [40] was used to obtain MREIT data in the human subject. To minimize the noise standard deviation in multi-echo MR images, *l* = 1, 2, …, *N*_*E*_ measured *B*_*z*_ data were calculated as a weighted sum of individual echoes $B_{z,l}^{m},l=1,2,\ldots ,N_{E}$, where *N*_*E*_ represents the total number of echoes, via

$$\begin{array}{c} B_{z}^{m}=\sum_{l=1}^{N_{E}}W_{l}B_{z,l}^{m} \end{array}$$ (17)

The weighting factors $W_{l}$ were computed using [40]

$$\begin{array}{c} W_{l}=\frac{{\left(\rho_{l}T_{c,l}\right)}^{2}}{\sum_{k=1}^{N_{E}}{\left(\rho_{k}T_{c,k}\right)}^{2}} \end{array}$$ (18)

where *ρ*_*l*_ is the MR magnitude image of the *l*^*th*^ echo.

### Reconstruction of current density from measured magnetic flux density

Inside Ω, the current density **J** = (*J*_*x*_, *J*_*y*_, *J*_*z*_) and magnetic flux density **B** = (*B*_*x*_, *B*_*y*_, *B*_*z*_) are related by Ampere’s law

$$\begin{array}{c} J=\frac{1}{\mu_{0}}\nabla \times B. \end{array}$$ (19)

Direct calculation of **J** using Ampere’s law requires knowledge of all three components of **B**. Instead of measuring the full magnetic flux density vector, we instead estimated the projected current density **J**^*P*^ from $B_{z}^{m}$ alone, with reference to a computational model of the whole domain, Ω constructed using structural T1-weighted images. The model was assigned a uniform conductivity and used to generate a predicted current density distribution **J**_0_, including an estimate of the *z*-component of the current density. The projected current density was then calculated via [15]

$$\begin{array}{c} J^{P}=J_{0}+(\frac{\partial \psi }{\partial y},-\frac{\partial \psi }{\partial x},0) \end{array}$$ (20)

where *ψ* was estimated from the measured *B*_*z*_ via [15]

$$\begin{array}{c} \left\{ \begin{array}{c} \nabla_{xy}^{2}\psi =\frac{1}{\mu_{0}}\nabla^{2}B_{z}^{m}\;\;\text{in}\;\Omega_{t}\\ \psi =0\;\;\;\;\;\;\;\;\;\;\;\;\;\;\;\;\;\;\text{on}\;\partial \Omega_{t}. \end{array}\right. \end{array}$$ (21)

In (21), $\nabla_{xy}^{2}≔(\frac{\partial^{2}}{\partial x^{2}}+\frac{\partial^{2}}{\partial y^{2}})$ is the two-dimensional Laplacian operator. Uniform-conductivity numerical models used to compute **J**_0_ data were constructed using the shape and dimensions of the object found from structural MRI images. These models were then used to solve the Laplace equation, subject to the same boundary conditions *g* as in experiments, using the COMSOL-MATLAB interface (MLI, COMSOL Inc, Burlington, MA, USA) using

$$\begin{array}{c} \left\{ \begin{array}{c} \nabla \cdot (C_{0}\nabla u_{0})=0\;\text{in}\;\Omega \\ -C_{0}\nabla u_{0}\cdot n=g\;\text{on}\;\partial \Omega . \end{array}\right. \end{array}$$ (22)

where *u*_0_ is the voltage distribution caused by the external current injection *g*, and **C**_0_ represents an homogeneous and isotropic conductivity distribution.

In the human body, the measured magnetic flux density data suffers because of weak MR signals recovered from bone and skin regions, mainly due to their rapid T_2_ decay. Inclusion of such regions cause severe artifacts in reconstructed scale factor images [41]. In the human head studies used in this paper, we therefore reconstructed the regional projected current density, $J_{R_{t}}^{P}$ only inside the brain region, $R_{t}\subset \Omega_{t}$ (Fig 4 (b)) using the expression

$$\begin{array}{c} J_{R_{t}}^{P}=J_{0}{|}_{R_{t}}+(\frac{\partial \psi_{R_{t}}}{\partial y},-\frac{\partial \psi_{R_{t}}}{\partial x},0) \end{array}$$ (23)

where $J_{0}{|}_{R_{t}}$ denotes the solution of the Laplace equation in (22), restricted to $R_{t}$, and the quantity $\psi_{R_{t}}$ was estimated from the measured $B_{z}^{m}$ and simulated *B*_*z*,0_ via [41]

$$\begin{array}{c} \left\{ \begin{array}{c} \nabla_{xy}^{2}\psi_{R_{t}}=\frac{1}{\mu_{0}}\nabla^{2}B_{z}^{m}\;\;\;\;\;\;\;\;\;\text{in}\;R_{t}\\ \psi_{R_{t}}=\frac{1}{\mu_{0}}(B_{z}^{m}-B_{z,0})\;\;\text{on}\;\partial R_{t}. \end{array}\right. \end{array}$$ (24)

Note that comparing the equation in (21) with (24) only differs in boundary which stems from the fact that for a simply connected local region the normal component of the current density is continuous [41].

### Simulation of current density and magnetic flux density data

In calculating training data from computational models of segmented MR magnitude data, it is necessary to determine both the predicted current density and the *z*-component of the magnetic flux density, *B*_*z*_, caused by the current flow in Ω at any position **r** in three dimensional space. Current density data were calculated using COMSOL via the methods described for determining **J** above, but with the conductivity distribution determined using standard segmentation of brain tissues and literature-estimated conductivity values. The magnetic flux density distribution was then computed from simulated **J** data using the Biot-Savart law

$$\begin{array}{c} B_{z}\left(r\right)=\frac{\mu_{0}}{4\pi }\int_{\Omega }\frac{\left(y-y^{{}^{\prime }}\right)J_{x}\left(r^{{}^{\prime }}\right)-\left(x-x^{{}^{\prime }}\right)J_{y}\left(r^{{}^{\prime }}\right)}{|r-r^{{}^{\prime }}{|}^{3}}dr^{{}^{\prime }} \end{array}$$ (25)

where *μ*_0_ is the magnetic permeability of free space. A fast Fourier transform implementation of (25) was used to improve calculation speeds [42]. The Biot-Savart law was used to calculate $B_{z}^{*}$ and ${\tilde{B}}_{z}$ images required for training data sets (Fig 2). Magnetic flux density data derived from uniform models (*B*_*z*,0_) were also employed for reconstructions of current density in (24) used in estimations of projected current densities used for generating both training and input data for the GRNN.

### Reconstruction of diffusion tensor data

Diffusion tensor volumes were reconstructed following Basser *et al*. [30], by comparing the diffusion weighted signal, $S_{b}^{j}$ with the non-diffusion weighted signal *S*_0_ to estimate

$$\begin{array}{c} {\left(g^{j}\right)}^{T}Dg^{j}=-\frac{1}{b}\ln(\frac{S_{b}^{j}}{S_{0}}) \end{array}$$ (26)

where, $g^{j}={\left[g_{x}^{j},g_{y}^{j},g_{z}^{j}\right]}^{T}$, *j* = 1, 2, …, *N_d_* are the normalized magnetic field gradient vectors of diffusion gradients applied in the sequence. Before estimating the diffusion tensor, any susceptibility-related geometric distortion caused by the EPI diffusion weighted imaging sequence was corrected [8, 43]. Six gradient directions were used in measuring phantom diffusion data, and 15 directions were employed in measuring diffusion properties of the human subject.

### Generation of training data sets

To generate the training data sets U and V, we constructed models containing piecewise-constant *η* values for each distinct tissue, and solved a total of M forward models with different discrete combinations of *η* values using the experimental montage $E_{m}$. This process produced M sets of simulated *B*_*z*_ data, denoted $B_{z}^{*}$, using the methods described above.

We additionally simulated results from a complementary montage, $E_{c}$ (shown in Fig 2) to generate a second set of M *B*_*z*_ data sets, ${\tilde{B}}_{z}$, using the same current injection amplitude as in experiments. The complementary montage model in each case differed only in the position of the two electrodes. These complementary positions were chosen to ensure current flow was not collinear with that caused by $E_{m}$, so that a unique conductivity reconstruction could be produced with the two-current DT-MREIT algorithm [6]. Noise was added independently to both $B_{z}^{*}$ and ${\tilde{B}}_{z}$, depending on the noise levels measured in experimental data $B_{z}^{m}$ for each imaging experiment. A common isotropic *η* distribution, designated ${\tilde{\eta }}^{*}$, was reconstructed from noisy $B_{z}^{*}$ and ${\tilde{B}}_{z}$ to obtain $V\in R^{P\times M}$ artifact free data using the two-current injection DT-MREIT algorithm [9, 10, 19, 33].

Two independent current densities J* and $\tilde{J}$ were calculated using Eqs (20) (or (23)) for the experimental $E_{m}$ and complementary $E_{c}$ electrode montages, respectively.

The curl-free property of the electric field leads to the following equation [9, 10, 19, 33]

$$\begin{array}{c} \nabla \ln\left({\tilde{\eta }}^{*}\right)\times (D^{-1}J)=\nabla \times (D^{-1}J). \end{array}$$ (27)

This equation can be re-expressed in matrix form as

$$\begin{array}{c} \tilde{A}e=\tilde{b} \end{array}$$ (28)

where $e={\left[\frac{\partial \ln({\tilde{\eta }}^{*})}{\partial x},\frac{\partial \ln({\tilde{\eta }}^{*})}{\partial y}\right]}^{T}$, ln quantities were calculated relative to 1 *S* ⋅ *sec*/*mm*^3^, and the matrices $\tilde{A}$ and $\tilde{b}$ are

$$\begin{array}{c} \begin{array}{c} \tilde{A}=(\begin{array}{cc} w_{1}{\left(D^{-1}J^{*}\right)}_{y}\left(x_{1},y_{1}\right) & \;-w_{1}{\left(D^{-1}J^{*}\right)}_{x}\left(x_{1},y_{1}\right)\\ w_{1}{\left(D^{-1}\tilde{J}\right)}_{y}\left(x_{1},y_{1}\right) & \;-w_{1}{\left(D^{-1}\tilde{J}\right)}_{x}\left(x_{1},y_{1}\right)\\ \;\;\;\vdots & \;\;\;\;\;\vdots \\ w_{N}{\left(D^{-1}J^{*}\right)}_{y}\left(x_{N},y_{N}\right) & \;-w_{N}{\left(D^{-1}J^{*}\right)}_{x}\left(x_{N},y_{N}\right)\\ w_{N}{\left(D^{-1}\tilde{J}\right)}_{y}\left(x_{N},y_{N}\right) & \;-w_{N}{\left(D^{-1}\tilde{J}\right)}_{x}\left(x_{N},y_{N}\right) \end{array})\;\text{and}\\ \tilde{b}=(\begin{array}{c} w_{1}(\frac{\partial {\left(D^{-1}J^{*}\right)}_{y}}{\partial x}-\frac{\partial {\left(D^{-1}J^{*}\right)}_{x}}{\partial y})\left(x_{1},y_{1}\right)\\ w_{1}(\frac{\partial {\left(D^{-1}\tilde{J}\right)}_{y}}{\partial x}-\frac{\partial {\left(D^{-1}\tilde{J}\right)}_{x}}{\partial y})\left(x_{1},y_{1}\right)\\ \;\;\;\;\;\;\vdots \\ w_{N}(\frac{\partial {\left(D^{-1}J^{*}\right)}_{y}}{\partial x}-\frac{\partial {\left(D^{-1}J^{*}\right)}_{x}}{\partial y})\left(x_{N},y_{N}\right)\\ w_{N}(\frac{\partial {\left(D^{-1}\tilde{J}\right)}_{y}}{\partial x}-\frac{\partial {\left(D^{-1}\tilde{J}\right)}_{x}}{\partial y})\left(x_{N},y_{N}\right) \end{array}) \end{array}. \end{array}$$ (29)

The weights *w*_*i*_ in Eq (29) relate to the MR-magnitude *ρ* and the noise level *h* associated with the measured noise (Fig 2) [33] as

$$\begin{array}{c} w_{i}=\frac{e^{-h||\rho \left(x_{i},y_{i}\right)-\rho \left(x,y\right)\left|\right|}}{\sum_{J=1}^{N}e^{-h||\rho \left(x_{j},y_{j}\right)-\rho \left(x,y\right)\left|\right|}}. \end{array}$$ (30)

In all reconstructions, a 3 × 3 window was used to determine the stiff matrix $\tilde{A}$ and load vector $\tilde{b}$ (29). Singular value decomposition was then used to decompose the 2*N* × 2 stiff matrix $\tilde{A}$ into *UΣV*^*T*^, where the two singular values of $\tilde{A}$ are entries in *Σ* = *diag*(*s*_1_, *s*_2_) and $\hat{b}=U^{T}\tilde{b}$.

The distribution **e** was solved for by minimizing the generalized cross-validation (GCV) function [32]

$$\begin{array}{c} GCV\left(\zeta \right)=\frac{\sum_{i=1}^{2}{\left(\frac{{\hat{b}}_{i}^{2}}{s_{i}^{2}+\zeta }\right)}^{2}}{{\left(\sum_{i=1}^{2}\frac{1}{s_{i}^{2}+\zeta }\right)}^{2}}. \end{array}$$ (31)

Here, *ζ* is a regularization constant estimated from the region $N_{\left(x,y\right)}=\left\{\left(x_{i},y_{i}\right)\in 1,2,3,\ldots N\right\}$ around the voxel position (*x*, *y*).

Finally, the ${\tilde{\eta }}^{*}$ images were obtained from **e** values by solving the two-dimensional Poisson Eq (32) assuming a known boundary value ${\tilde{\eta }}_{\partial \Omega }^{*}$ [10, 20, 33]

$$\begin{array}{c} \left\{ \begin{array}{cc} \nabla_{xy}^{2}\ln\left({\tilde{\eta }}^{*}\right)=\nabla_{xy}\cdot e & \text{in}\;\Omega_{t}\\ \ln\left({\tilde{\eta }}^{*}\right)=\ln\left({\tilde{\eta }}_{\partial \Omega }^{*}\right) & \text{on}\;\partial \Omega_{t}. \end{array}\right. \end{array}$$ (32)

Next, we reconstructed η* from simulated noisy $E_{m}$ data $B_{z}^{*}$ alone, using the dual-loop network, to create an input data set $U\in R^{P\times M}$. The two $R^{P\times M}$ data sets U and V were then used to build the network using the ‘newgrnn’ function implemented in the MATLAB Neural Network Toolbox. After generating the training data sets for each projection, one thousand hidden neurons were used to perform ANN correction, both for phantom and human experiments. We found that the training time for data used in this paper averaged around 30 minutes ysing a MacPro computer with an Intel(R) Xenon(R) (E5–2697 v2, 2.70 GHz) 12 core CPU and 64.0 GB RAM.

#### Phantom image training data

**Fig 3:**
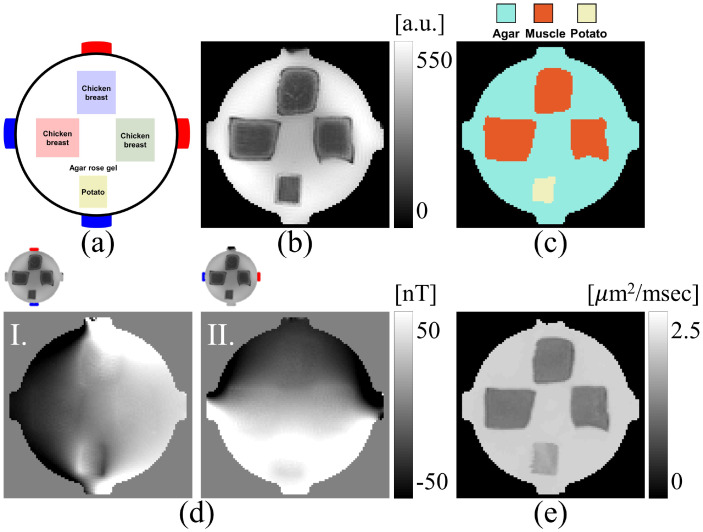
Phantom experiment setup. (a) Phantom design, (b) MR magnitude image from spin-echo pulse sequence during MREIT experiment, (c) Segmented ROIs for volume conductor model. The ROIs were segmented using the unsupervised data partitioning method implemented in the MATLAB command kmeans. (d) $B_{z}^{m,E},E=1\;\text{(vertical)}\;\text{and}\;\text{2}\;\text{(horizontal)}$ images induced due to the 10 mA current injection, (e) Mean diffusivity map obtained from six direction diffusion data sets with *b*-value 1000 *sec*/*mm*^2^. Images in (b)-(e) cropped to 100 × 100 pixels^2^ to show detail.

To calculate $B_{z}^{*}$ and $\tilde{B_{z}}$ distributions used to reconstruct phantom experiment data, we segmented tissue masks into three different ROIs, shown in Fig 3(c) for the center slice. Each of the three distinct tissues (agar, chicken, potato) were simulated at ten linearly spaced *η* values, resulting in a total of M=10×10×10=1000 models. Agar was simulated with *η* between 0.1 and 1 S ⋅s/mm^3^, chicken between 0.2 and 1.25 S ⋅ s/mm^3^ and potato between 0.01 and 0.3 S ⋅ s/mm^3^. Noise was added to $B_{z}^{*}$ and ${\tilde{B}}_{z}$ data independently, based on the SNR determined in the agar region of phantom magnitude images. From 16 we estimated the standard deviation of measured *B*_*z*_ data in phantom experiments was about 0.18 nT.

#### Human image training data

**Fig 4:**
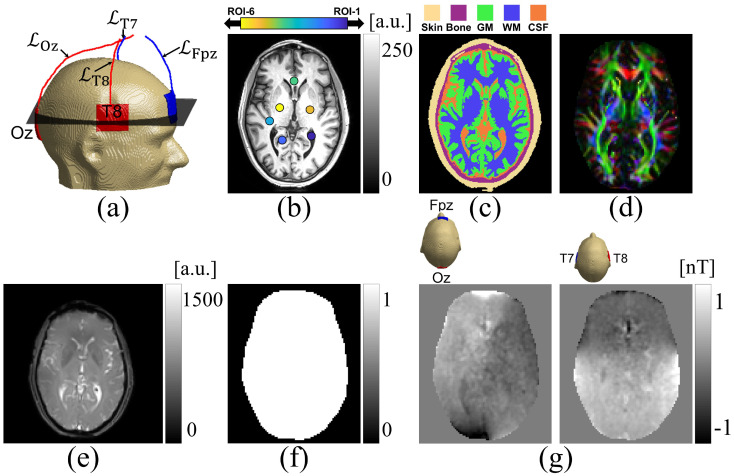
Human experiment set-up. (a) Sagittal view of computer model of human subject with attached electrodes (Fpz, Oz, T7, T8). The corresponding lead wire trajectories are marked as $L_{\text{Fpz}}$, $L_{\text{Oz}}$, $L_{\text{T7}}$, $L_{\text{T8}}$. (b) High resolution T_1_-weighted image corresponding to the MREIT slice shown in (e). Locations of six ROIs used for evaluation of reconstructed conductivity values shown in Table 5. (c) Segmented tissues in volume conductor model. Tissue segmentation was performed using the method described in Huang *et al*. [50]. (d) Colour-coded fractional anisotropy map obtained from fifteen-direction diffusion data sets with *b*-value 1000 *sec*/*mm*^2^. (e) MR magnitude image from multi-gradient multi-echo pulse sequence during MREIT acquisition. (f) Brain ROI ($R_{t}$), mask used in electromagnetic field reconstruction. (c) Echo-combined $B_{z}^{m,E},E=1\;\text{(Fpz-Oz),}\;\text{and}\;\text{2}\;\text{(T7-T8)}$ images induced due to the 1.5 mA current injection. Calculated stray magnetic fields were subtracted from individual echo images before further processing. Images in (b)-(c) are cropped to 175 × 210 and those in (d), (f)-(g) are cropped to 85 × 100.

In simulating scaling factors for the human experiment, we segmented tissue masks into five different ROIs, as shown in Fig 4(e) for the center slice. We assumed skin and bone regions had isotropic conductivity values of 0.43 S/m [29] and 0.015 S/m [44] respectively. The brain region, consisting of gray matter (GM), white matter (WM) and CSF was modeled by scaling the measured diffusion tensor [31, 45]. A total of M=10×10×10=1000 sets of numerical model data were generated by independently varying model scale factor values for GM between 0.210 and 0.736 S ⋅ s/mm^3^ [46], for WM between 0.210 and 0.736 S ⋅ s/mm^3^ [46], and for CSF between 0.700 and 0.844 S ⋅ s/mm^3^ [31, 45] respectively. Table 1 summarizes the range of effective isotropic conductivity values calculated from the conductivity tensor used to generate numerical model data. As for phantom data noise was added to $B_{z}^{*}$ and ${\tilde{B}}_{z}$ data. The SNR determined in the white matter region of magnitude images was used to estimate noise levels for human data. Noise standard deviations of 0.21 nT were added to *B*_*z*_ data for the human imaging experiment.

**Table 1:** Range of isotropic conductivity values used to generate numerical model data. Isotropic conductivities were estimated using $\sqrt{C_{l}C_{t}}$ [29], where *C*_*l*_ = λ_*C*,1_ and *C*_*t*_ = (λ_*C*,2_ + λ_*C*,3_)/2 are the longitudinal and tranversal components of the conductivity, respectively and λ_*C*,1_ ≥ λ_*C*,2_ ≥ λ_*C*,3_.

Model	Tissues	Isotropic conductivity [S/m]	Model	Tissues	Isotropic conductivity [S/m]
Phantom	Chicken	0.18–2.67	Human	GM	0.02–1.32
	Potato	0.02–0.60		WM	0.01–3.27
	Agar	0.10–2.13		CSF	0.71–3.75

### Electric field reconstruction

In some cases, electric field images were constructed from reconstructed current density and conductivity tensor data. Using the reconstructed projected current density, $J^{P,E}$ or $J_{R_{t}}^{P,E}$ and estimated conductivity tensor **C**, electric field vectors at locations **r** within images were reconstructed as

$$\begin{array}{c} E^{E}\left(r\right)=C^{-1}\left(r\right)J^{P,E}\left(r\right) \end{array}$$ (33a)

$$\begin{array}{c} E_{R_{t}}^{E}\left(r\right)=C^{-1}\left(r\right)J_{R_{t}}^{P,E}\left(r\right) \end{array}$$ (33b)

### Verification of reconstruction performance

To evaluate the proposed one-current reconstruction method, a common isotropic scale factor distribution, designated $\tilde{\eta }$, was also reconstructed from experimentally measured projected current density ($J^{P,E},E=1,2$) data, by solving Eqs (31) and (32) using the known boundary scaling factor ${\tilde{\eta }}_{\partial \Omega }$ [20].

Dual-loop results corrected by the ANN were verified against DT-MREIT reconstructions and also against known conductivity values, where available. Dual-loop and DT-MREIT reconstructions were compared using a relative *L*^2^ measure, where, for any reference or true data x and reconstructed data y, the relative *L*^2^-difference or error *RE* defined as

$$\begin{array}{c} RE=\frac{∥x-y∥}{∥x∥}. \end{array}$$ (34)

Here, ‖⋅‖ represents the Euclidean distance.

We also measured the structural similarity (SSIM) of reconstructions [47]. The structural similarity measures the similarity between a true or reference image x and an input or reconstructed image y by comparing the luminance, contrast and their structures

$$\begin{array}{c} SSIM\left(x,y\right)=\frac{\left(2\mu_{x}\mu_{y}+C_{1})(2\sigma_{xy}+C_{2}\right)}{\left(\mu_{x}^{2}+\mu_{y}^{2}+C_{1})(\sigma_{x}^{2}+\sigma_{y}^{2}+C_{2}\right)} \end{array}$$ (35)

Here, $\mu_{x}$, $\mu_{y}$, $\sigma_{x}$, and $\sigma_{y}$ are the local mean and standard deviations of the image x and y respectively and *C*_1_ and *C*_2_ are two regularization constants chosen to avoid instability when the denominator of the expression is close to zero [47]. A 5 × 5 window was used to calculate means and standard deviations and *C*_1_ and *C*_2_ values were set to be 1 × 10^−4^ and 9 × 10^−4^ respectively. The mean SSIM value was then computed as

$$\begin{array}{c} MSSIM=\frac{1}{P_{s}}\sum_{j=1}^{P_{s}}SSIM_{j}\left(x,y\right) \end{array}$$ (36)

where $P_{s}$ is the total number of non-zero voxels in the image slice.

### Phantom experiment description

A phantom experiment was conducted to test the proposed method. The cylindrical phantom had a 55 mm diameter and was 50 mm high. Two opposing pairs of 10 × 10 *mm*^2^ carbon electrodes (Hurev Co. Ltd., South Korea) were attached to the perimeter as shown in Fig 3(a). Three cubes of chicken muscle (∼15 × 15 × 15 *mm*^3^) oriented in *x* (left), *y* (right), *z*-directions (top) shown in were placed inside the phantom, centered on the electrode plane. A small cube of potato (∼8 × 8 × 15 *mm*^3^), assumed isotropic, was also placed inside the phantom. The background of the phantom was filled with a 1.0 S/m conductive gel made with agarose.

All data were measured using a single-channel RF volume coil in a 7.0 T Bruker scanner (Bruker Biospin MRI, Billerica, MA, USA) located at the Barrow Neurological Institute (Phoenix, Arizona, USA). A custom-designed constant current source [48] was used to deliver 10 mA current synchronously with spin-echo MREIT acquisitions with a total current injection time *T*_*c*_, of 20 ms in each TR. Two sets of $B_{z}^{m,E}\;E=1,2$ data were collected over five slices, with an image matrix size of 128 × 128. Other imaging parameters were, TR/TE = 1000/20 ms, field-of-view, FOV = 80 × 80 *mm*^2^, slice thickness, 3 mm (no slice gap), number of excitation, NEX = 8, and number of echoes, *N*_*E*_ = 1. *B*_*z*_ images were calculated using Eq (15). Fig 3(b) and 3(d) shows the acquired MR magnitude and $B_{z}^{m,E},\;E=1\;\text{(`vertical')}\;\text{and}\;\text{2}\;\text{(`horizontal')}$ images respectively for the center slice.

Diffusion tensor data of the same five slices were then collected using a single-shot spin-echo echo planar imaging (SSSE-EPI) pulse sequence with *b* *of* 1000 *sec*/*mm*^2^ and a total of six diffusion directions. Data were acquired with TR/TE = 2500/32.67 ms. Other parameters were the same as for MREIT image sequences. One reference data set with *b* = 0 *sec*/*mm*^2^ was also collected. The diffusion tensor distribution was reconstructed using (26). The condition number of the DTI scheme was 4.7. Therefore, prior to reconstruction the diffusion weighted data set was denoised by taking local features of the diffusion weighted images into account, using an adaptive Wiener filter [49] implemented in MATLAB (https://www.mathworks.com/matlabcentral/fileexchange/43992).

### Human experiment description

Human experimental protocols were approved by the Arizona State University institutional review board and all procedures were carried out in accordance with these protocols after obtaining informed consent from participants. Data obtained from a 58-year old volunteer male human subject were used to demonstrate the performance of the proposed method. Data were acquired using a 32-channel RF head coil and a 3.0T Phillips scanner (Phillips, Ingenia, Netherlands) located at the Barrow Neurological Institute (Phoenix, Arizona, USA). A transcranial electrical stimulator (DC-STIMULATOR MR, neuroConn, Ilmenau, Germany) was used to deliver 1.5 mA currents using both Fpz-Oz and T8-T7 electrode montages. Sets of $B_{z}^{m,E}\;E=1,2$ data were measured over three axial slices using a multi-echo-gradient-echo pulse sequence and an image matrix size of 128 × 128. Other imaging parameters were, TR/TE/ES = 50/7/3 ms, field-of-view, FOV = 224 × 224 *mm*^2^, slice thickness, 5 mm (no slice gap), number of excitations NEX = 24, and number of echoes, *N*_*E*_ = 10. The current injection time *T*_*c*_ in each TR was 32 ms. *B*_*z*_ images of each echo were calculated using Eq (15). Individual echo images were then combined using (17) to improve the SNR of the acquired *B*_*z*_ signals. Prior to echo combination, stray magnetic field corrections were applied to each echo image [51]. Fig 4(e) and 4(g) show acquired MR magnitude and $B_{z}^{m,E},\;E=1\;\text{(`Fpz-Oz'),}\;\text{and}\;\text{2}\;\text{(`T7-T8')}$ images respectively for the center slice.

Diffusion-weighted images of the three MREIT slices were also acquired, using a single-shot, spin-echo-echo-planar (SE-EPI) imaging sequence, with *b* = 1000 *sec*/*mm*^2^ and a total of fifteen diffusion directions. The condition number of the DTI scheme was 1.3. Data were acquired with TR/TE = 2000/139 ms and *NEX* = 2. The acquisition matrix size was 64 × 64, with the same FOV as in MREIT sequences. The data were then interpolated to 128 × 128 matrix to match the MREIT data. One reference data set with *b* = 0 *sec*/*mm*^2^ was also collected.

Parameters used in both phantom and human experiments are summarized in Table 2. For each experiment, diffusion data were co-registered with T_1_ weighted MR-images, and diffusion tensors were reconstructed using Eq (26). ure Fig 3(e) shows the mean diffusivity map ($\frac{D_{xx}+D_{yy}+D_{zz}}{3}$) estimated from the denoised diffusion weighted images for the phantom, and Fig 4(d) shows the colour-coded fractional anisotropy map estimated from the diffusion weighted images for the human subject. In the human experiment, *B*_*z*_ data were corrected to take into account any stray magnetic field [51, 52] created by lead wires that may have affected phase images. For the phantom experiment, we estimated **J**^*P*^ (Eq (20)) using the Laplacian (∇^2^) of the measured $B_{z}^{m}$ data (Eq (21)). Therefore, it was not necessary to consider wire-related stray magnetic fields in the phantom data because $\nabla^{2}B_{z,L}\left(r\right)=0\;\text{in}\;r\in \Omega$ [6]. However, for the human experiment, the regional projected current density in Eq (23) [41] was estimated using the local boundary condition (24). In this case the stray magnetic field must be accounted for. Therefore, a numerical model of the wires in the human experiment was constructed from 1 mm^3^-resolution T_1_-weighted images in order to estimate these stray magnetic fields via the Biot-Savart law (see S1 File).

**Table 2:** Experimental image parameters.

Experiment	Pulse sequence	Resolution (mm^3^)	FOV (mm^3^)	TR/TE/ES (ms)	*N*_*E*_/*NAS*
MREIT(phantom-1)	SE (7T Bruker), 10mA current	0.625 × 0.625 × 3	80 × 80 × 15	1000/20	1/8
MREIT(*in vivo* human)	mffe (3T Phillips), 1.5 mA current	1.75 × 1.75 × 5	224 × 224 × 15	50/7/3	10/24
MREIT(phantom-2)	SE (7T Bruker), 10 mA current	1 × 1 × 4	64 × 64 × 28	1000/20	1/2
DWI(phantom-1)	SS-SE-EPI(7T Bruker *b* = 1000 s/mm^2^, 6 directions)	0.625 × 0.625 × 3	80 × 80 × 15	2500/32.67	1/2
DWI(*in vivo* human)	SS-SE-EPI(3T Phillips *b* = 1000 s/mm^2^, 15 directions)	3.5 × 3.5 × 5	224 × 224 × 15	2000/139	1/2
DWI(phantom-2)	SS-SE-EPI(7T Bruker *b* = 1000 s/mm^2^, 6 directions)	1 × 1 × 4	64 × 64 × 28	2300/27	1/2

## Results

### Phantom experiment results

**Fig 5:**
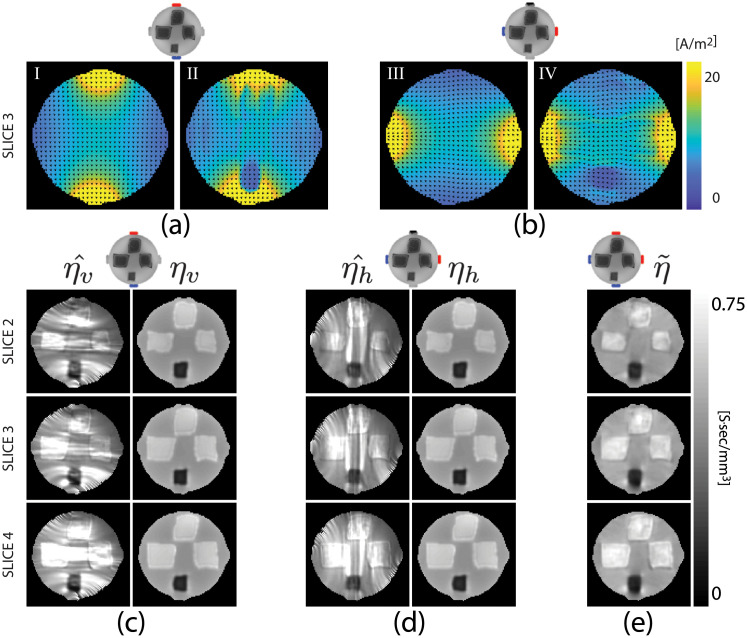
Model-predicted current density images for the central slice (slice 3) of the phantom obtained from a homogeneous (I, III) numerical model and estimated current density images found using Eq (20) (II, IV) for (a) vertical and (b) horizontal current injection. Normalized arrow plots overlaid on images show current flow directions. Parts (c) and (d) illustrate reconstructed scale factor images of the three central slices (2, 3, 4) of the phantom object for vertical (c) and horizontal (d) currents. $\hat{\eta }$ images in parts (c) and (d) are solutions of the dual-loop matrix system (9). *η*_*v*,*h*_ images show ANN-corrected scale factors. Part (e) ($\tilde{\eta }$) shows scale factor images recovered from data measured using two current injections. Images cropped to 95 × 95.

ure Fig 5(a) and 5(b) shows reconstructed projected current density magnitudes in the biological tissue phantom. Images were calculated using Eq (20).

Reconstructed scale factor images solving Eq (9) estimated from measured current density and diffusion tensor data are displayed as ${\hat{\eta }}_{v}$ and ${\hat{\eta }}_{h}$ in Fig 5(c) and 5(d) for vertical and horizontal currents, respectively. Images labeled *η*_*v*_ and *η*_*h*_ in Fig 5(a) and 5(b) show ANN-corrected scale factor images obtained from the trained neural network. The optimum spread-constant values were found to be 0.75 for both vertical- (E=1) and horizontal- (E=2) current networks. ure Fig 5(e) shows the reconstructed scale factor images ($\tilde{\eta }$) using the two-current injection DT-MREIT algorithm. We also computed the relative *L*^2^-differences and measured the similarity index [47] of these reconstructed scale factor images. For the vertical projection, relative *L*^2^-differences were found to be 0.11, 0.12 and 0.12 for the second, third and fourth slices, respectively, after ANN correction. Similarly, using the single-current dual loop estimation for the horizontal current injection, relative *L*^2^-differences were calculated to be 0.11, 0.12 and 0.12. Similarity indices for vertical current injections were found to be 0.94 in each of the second, third and fourth slice positions after ANN correction. For the single-current dual loop estimation from horizontal current injection, similarity indices for these three slices were also found to be 0.94.

**Fig 6:**
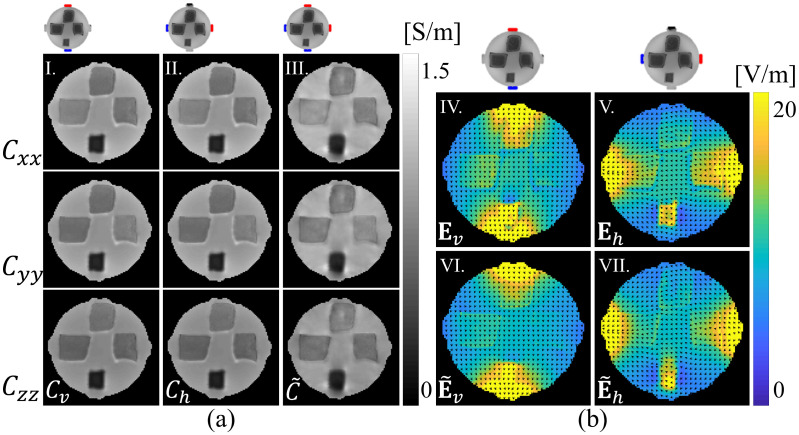
Reconstructed conductivity tensor and electric field comparisons for central slice. Part (a) compares of reconstructed conductivity tensor using proposed one-current injection method with the two-current injection DT-MREIT algorithm [33] using vertical injection (**C**_*v*_) and horizontal injection (**C**_*h*_) only or ($\tilde{C}$) using the two-current injection method. (b) Electric field maps derived from reconstructed conductivity tensors of the central phantom slice. Images labeled **E**_*v*_ and **E**_*h*_ show electric field magnitudes estimated from reconstructed conductivity tensors **C**_*v*_ and **C**_*h*_ respectively. Images ${\tilde{E}}_{v}$ and ${\tilde{E}}_{h}$ show estimated electric field distributions for horizontal and vertical current injection, respectively, from conductivity tensor reconstructed from two-injection current injection $\tilde{C}$. The normalized arrow plots overlaid on (b) show **E** field directions. Images cropped to 95 × 95.

Reconstructed scale factors were multiplied with diffusion tensors to produce conductivity tensors as in (3). Results for the center (third) slice are shown in Fig 6(a). Fig 6(a) shows reconstructed diagonal components of the conductivity tensor found using the ANN-corrected single-current injection for vertical (*C*_*v*_) and horizontal (*C*_*h*_) projections, respectively, and Fig 6(a) also displays the conductivity tensor $\tilde{C}$ found using the two-current DT-MREIT for the center slice. Actual reconstructed conductivity values and anisotropic ratio, $AR=\frac{2\lambda_{C,1}}{\lambda_{C,2}+\lambda_{C,3}}$, where λ_*C*,*k*_ *k* = 1, 2, 3 are the three eigenvalues of the reconstructed conductivity tensor, are summarized in Table 3. Note that due to the scaled relationship of the conductivity and water diffusion tensors, anisotropic ratios in reconstructed conductivity tensors were the same as in water diffusion tensors. Therefore, these values are identical for all three reconstructions compared in Table 3. The proposed one-current injection method showed good agreement with the two-current DT-MREIT reconstructions. However, the anisotropic ratio, which was directly derived from diffusion weighted data sets, was small for all five ROIs, especially for the top (*z*-oriented) chicken muscle tissue sample, where the *C*_*zz*_ conductivity component was expected to be largest, along with the anisotropic ratio.

**Table 3:** Reconstructed diagonal components of the conductivity tensor (S/m) and anisotropic ratio *AR* for biological tissue phantom images in Fig 6. Underlined entries show tensor components along tissue orientation directions.

		Agar	Left tissue	Right tissue	Top tissue	Bottom tissue
Vertical one-current	*C*_*xx*_	1.06 ± 0.03	0.85 ± 0.04	0.75 ± 0.03	0.75 ± 0.03	0.19 ± 0.01
	*C*_*yy*_	1.02 ± 0.03	0.74 ± 0.05	0.80 ± 0.03	0.72 ± 0.04	0.19 ± 0.01
	*C*_*zz*_	0.92 ± 0.03	0.68 ± 0.05	0.65 ± 0.03	0.73 ± 0.03	0.17 ± 0.01
Horizontal one-current	*C*_*xx*_	1.06 ± 0.03	0.85 ± 0.04	0.75 ± 0.03	0.75 ± 0.03	0.19 ± 0.01
	*C*_*yy*_	1.02 ± 0.03	0.74 ± 0.05	0.80 ± 0.03	0.72 ± 0.04	0.19 ± 0.01
	*C*_*zz*_	0.92 ± 0.03	0.68 ± 0.05	0.65 ± 0.03	0.73 ± 0.03	0.17 ± 0.01
DT-MREIT two-current	*C*_*xx*_	1.06 ± 0.03	0.89 ± 0.04	0.77 ± 0.03	0.77 ± 0.04	0.17 ± 0.02
	*C*_*yy*_	1.02 ± 0.03	0.75 ± 0.03	0.83 ± 0.03	0.75 ± 0.04	0.17 ± 0.02
	*C*_*zz*_	0.92 ± 0.03	0.67 ± 0.03	0.66 ± 0.03	0.76 ± 0.03	0.15 ± 0.02
	*AR*	1.11 ± 0.01	1.21 ± 0.06	1.15 ± 0.03	1.07 ± 0.03	1.11 ± 0.02

For comparison purposes, we also measured biological tissue conductivities at 100 Hz using an impedance analyzer and a four-probe method. These values showed good agreement with the reconstructed conductivity (Table 4). For example, the conductivity of the potato sample at 100 Hz was found to 0.18±0.006 S/m, an approximate 0.73% relative *L*^2^ error. Similarly, for the chicken breast muscle the measured impedances indicated a conductivity of 0.86±0.01, and 0.73±0.008 S/m along and across the fiber directions, respectively. For all three tissue samples the average conductivity value along the fibers was found to be (Table 4) 0.82 S/m, while those across fibers were 0.71 S/m, an approximate 2–4% error. For the agar background the relative *L*^2^ error was found to be 1.60% with respect to impedance analyzer conductivity measurements.

**Table 4:** Comparison of tissue conductivities measured (S/m) by an impedance analyzer using a four-probe method and conductivity values averaged over phantom regions of interest for chicken, potato or agar materials from the image shown in Fig 6, using the proposed method and the two-current injection DT-MREIT algorithm [33]. The longitudinal and transverse components of the conductivity, *C*_*l*_ = λ_*C*,1_ and *C*_*t*_ = (λ_*C*,2_+λ_*C*,3_)/2 were calculated from the eigenvalues (λ_*C*,1_≥λ_*C*,2_≥λ_*C*,3_) of the reconstructed conductivity tensor. Effective isotropic conductivities of potato and agar backgrounds were calculated using the relation, $C_{i}=\sqrt{C_{l}C_{t}}$ [29] for potato and agar regions for comparison with impedance analyzer measurements.

Method	Type	Chicken	Potato	Agar
Impedance analyzer	-	*C*_*l*_ = 0.86 ± 0.010	*C*_*i*_ = 0.18 ± 0.006	*C*_*i*_ = 1.00 ± 0.018
		*C*_*t*_ = 0.73 ± 0.008		
Proposed	Vertical	*C*_*l*_ = 0.81 ± 0.003	*C*_*i*_ = 0.19 ± 0.009	*C*_*i*_ = 1.02 ± 0.031
		*C*_*t*_ = 0.71 ± 0.010		
	Horizontal	*C*_*l*_ = 0.81 ± 0.003	*C*_*i*_ = 0.19 ± 0.009	*C*_*i*_ = 1.02 ± 0.031
		*C*_*t*_ = 0.71 ± 0.010		
DTMREIT two current	-	*C*_*l*_ = 0.84 ± 0.003	*C*_*i*_ = 0.17 ± 0.020	*C*_*i*_ = 1.02 ± 0.033
		*C*_*t*_ = 0.72 ± 0.002		

Fig 6(b) compares magnitudes of estimated vertical or horizontal electric fields found using Eq (33). The relative *L*^2^-differences between single-current injection and DT-MREIT reconstructed electric fields for vertical projection data were found to be 0.16, and 0.12 for the horizontal projection in the center slice shown in Fig 6(b).

### Human experiment results

**Fig 7:**
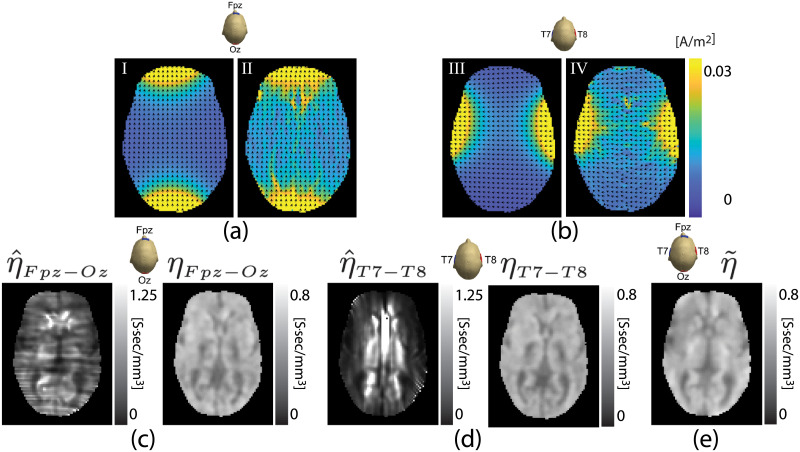
Model-predicted current density in a central slice of human head data $J_{0}{|}_{R_{t}}$ (I, III) obtained from Eq (22) and estimated current density images found using Eq (23) (II, IV) are shown for (a) Fpz-Oz and (b) T8-T7 electrode montages. Normalized arrow plots overlaid on images show current flow directions. Reconstructed scale factor images of the central slice of *in-vivo* human subject are shown in parts (c) and (d) for Fpz-Oz or T8-T7 current injections respectively. Images in (c) and (d) labeled $\hat{\eta }$ show solutions of the dual-loop matrix system in (9). Images labeled *η* show ANN-corrected scale factors. Part (e) shows a scale factor image $\tilde{\eta }$ recovered from data measured from both current injections. Images cropped to 75 × 100.

Fig 7(a) and 7(b) shows the magnitude of the reconstructed current density for Fpz-Oz and T8-T7 current injections respectively. Reconstructed scale factor images $\hat{\eta }$ solving Eq (9) estimated from measured current density and diffusion tensor data are displayed in Fig 7(c) and 7(d) for Fpz-Oz and T8-T7 currents respectively. Images labeled *η* in Fig 7 show ANN-corrected scale factor images obtained from the trained neural network. The $\hat{\alpha }$ values were found 1.85 for Fpz-Oz and 1.03 for the T7-T8 electrode montage, respectively.

Fig 7(e) shows a reconstructed scale factor image found using the two-current-injection DT-MREIT algorithm. We computed relative *L*^2^-differences and similarity indices for scale factor images reconstructed the two methods. For the Fpz-Oz and T7-T8 electrode montages, relative *L*^2^-differences and similarity indices were found to be 0.14 and 0.93, respectively after ANN correction.

**Fig 8:**
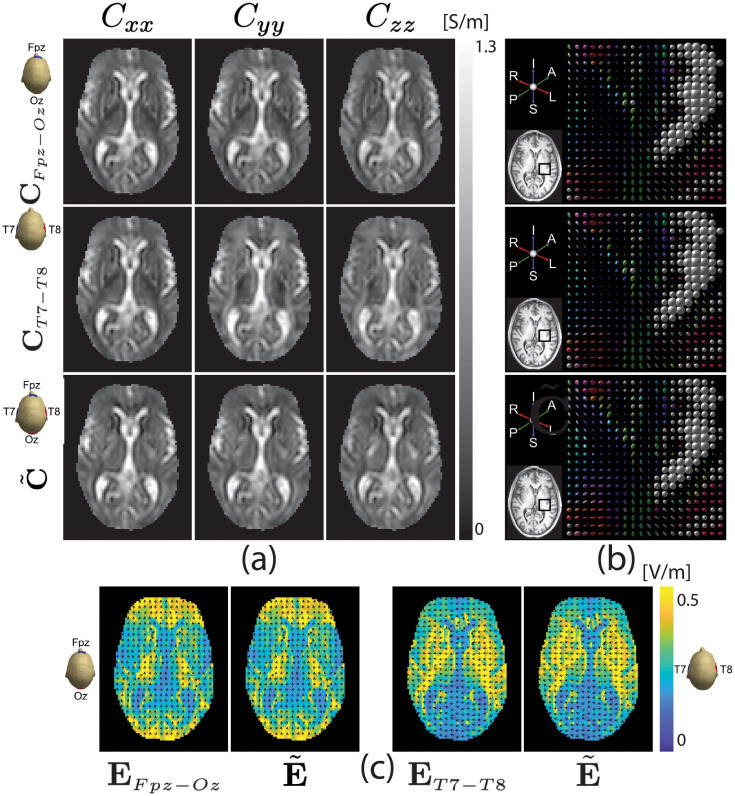
Reconstructed conductivity tensor and electric field comparisons for *in-vivo* human experiment. (a) Comparison of reconstructed conductivity tensor using proposed one-current injection method with the two-current injection DT-MREIT algorithm [33] using Fpz-Oz injection (**C** *_Fpz−Oz_*) and T8-T7 injection only (**C** *_T7−T8_*) or using the two-current injection method ($\tilde{C}$). Part (b) shows tensor plots of the reconstructed conductivity images in (a). The ROI is marked in the inset T_1_-weighted image. The size of each ellipsoid in (b) is proportional to the tensor eigenvalues at that location, and its color and orientation represent principal eigenvectors. Part (c) shows electric field maps derived from reconstructed conductivity tensors of the central slice, with the Fpz-Oz image at left and T8-T7 montage at right. Images labeled **E** denote electric field magnitudes estimated from reconstructed conductivity tensors shown in (a). Images labeled $\tilde{E}$ are estimated electric field distributions for were found from conductivity tensors reconstructed from the two-injection current injection shown in (a). Normalized arrow plots overlaid on (c) show **E** field directions. Reconstructed images cropped to 75 × 100.

Reconstructed scale factors were multiplied with diffusion tensors to produce conductivity tensors as in (3). Results for the center slice are shown in Fig 8(a). The top and middle rows of Fig 8(a) show reconstructed diagonal components of the conductivity tensor using ANN-corrected single-current injection for Fpz-Oz and T8-T7 projections, respectively, and the bottom row displays the conductivity tensor components found using the two-current DT-MREIT for the center slice. Fig 8(b) compares the reconstructed conductivity tensors within the selected ROI. Values for diagonal components of reconstructed conductivity tensors are summarized in Table 5. Fig 8(c) compares magnitudes of estimated Fpz-Oz or T8-T7 electric fields found using Eq (20). Relative *L*^2^ errors and *MSSIM* results for all phantom and human data slices are summarized in Table 6. The relative *L*^2^ difference between single-current injection and DT-MREIT reconstructed electric fields for the human subject Fpz-Oz projection data was found to be 0.13 for the center slice shown in Fig 8(c). For the same slice and T7-T8 data, the relative *L*^2^ difference in electric field was 0.09. Structural similarities to corresponding two-current-measured data for these electric field images were 0.94 and 0.98 respectively.

**Table 5:** Reconstructed diagonal components of the conductivity tensor (S/m) for *in-vivo* human experiment shown in Fig 8. The ROIs are displayed in Fig 4(d).

		ROI#1	ROI#2	ROI#3	ROI#4	ROI#5	ROI#6
Fpz-Oz	*C*_*xx*_	1.15±0.10	0.53±0.11	0.48±0.10	0.61±0.05	0.23±0.02	0.17±0.05
	*C*_*yy*_	1.14±0.10	0.55±0.09	0.48±0.10	0.26±0.06	0.55±0.03	0.22±0.08
	*C*_*zz*_	1.14±0.12	0.54±0.08	0.47±0.09	0.22±0.05	0.27±0.05	0.55±0.09
T8-T7	*C*_*xx*_	1.18±0.12	0.53±0.10	0.48±0.10	0.59±0.06	0.22±0.02	0.16±0.04
	*C*_*yy*_	1.18±0.11	0.54±0.07	0.48±0.10	0.25±0.06	0.53±0.05	0.21±0.10
	*C*_*zz*_	1.19±0.13	0.53±0.06	0.47±0.09	0.21±0.05	0.26±0.04	0.52±0.10
Fpz-Oz/T8-T7	*C*_*xx*_	1.20±0.11	0.58±0.11	0.40±0.08	0.55±0.06	0.22±0.02	0.21±0.03
	*C*_*yy*_	1.20±0.10	0.60±0.09	0.40±0.09	0.24±0.06	0.53±0.05	0.27±0.11
	*C*_*zz*_	1.23±0.11	0.59±0.07	0.39±0.08	0.20±0.05	0.26±0.05	0.73±0.09

**Table 6:** Relative *L*^2^-diferences (34) and mean structural similarity *MSSIM* indices (36) of reconstructed scale factor and E fields for proposed single-current and two-current injection DT-MREIT algorithms [33].

Study	Slice#	Current administration	Scale factor				E-field	
			Dual-loop only		Dual-loop with ANN correction		Dual-loop with ANN correction	
			*RE*	*MSSIM*	*RE*	*MSSIM*	*RE*	*MSSIM*
Phantom	2	Vertical	0.32	0.67	0.11	0.94	0.13	0.89
		Horizontal	0.29	0.75	0.11	0.94	0.11	0.93
	3	Vertical	0.29	0.72	0.12	0.94	0.16	0.92
		Horizontal	0.29	0.75	0.12	0.94	0.12	0.94
	4	Vertical	0.37	0.69	0.12	0.94	0.16	0.90
		Horizontal	0.27	0.77	0.12	0.94	0.15	0.93
Human	2	Fpz-Oz	0.35	0.62	0.14	0.93	0.13	0.94
		T7-T8	0.41	0.57	0.14	0.93	0.09	0.98

## Discussion

### Relationship between MREIT and DT-MREIT

We first note properties of the DT-MREIT reconstruction problem that are distinct from purely electromagnetic approaches. Without loss of generality, consider a three dimensional conductive domain Ω consisting of a background region Ω\R containing one anomaly R. The conductivity of the background is denoted $C_{b}$ and the anomaly conductivity is defined as **C** = **C** *_b_* + *δ* **C**. From fundamental electromagnetic principles [53], any point on the anomaly subdomain boundary, ξ∈∂R satisfies the relations

$$\begin{array}{c} \begin{array}{c} -C_{b}\nabla u^{+}\left(\xi \right)\cdot \nu =-\left(C_{b}+\delta C\right)\nabla u^{-}(\xi )\cdot \nu \\ \;\;\nabla u^{+}\left(\xi \right)\cdot \tau =\nabla u^{-}\left(\xi \right)\cdot \tau \end{array}\}\;\text{on}\;\xi \in \partial R \end{array}$$ (37)

where *ν* and **τ** are normal and tangential unit vectors on ∂R respectively, and the voltage distributions *u*^+^ and *u*^−^ are defined as

$$\begin{array}{c} u^{+}=u{{|}_{\;\Omega \backslash R}\;\;\text{and}\;\;u^{-}=u|}_{R}. \end{array}$$ (38)

Decomposing the current density vector **J** into normal and tangential part at the subdomain interface, **J**(*ξ*) = (**J**(*ξ*) ⋅ *ν*)*ν* + (**J**(*ξ*) ⋅ **τ**)**τ**, and using Eq (37) we have [34]

$$\begin{array}{c} J^{+}\left(\xi \right)-J^{-}\left(\xi \right)=(\delta C\nabla u^{-}\left(\xi \right)\cdot \tau )\tau \end{array}$$ (39)

where the current density vectors **J**^+^ and **J**^−^ are defined in the background and anomaly regions respectively in a similar manner to *u*^+^ and *u*^−^. However, in DT-MREIT the relationship **D**^−1^ **J** = −*η*∇*u* at the subdomain interface satisfies [18]

$$\begin{array}{c} \begin{array}{c} (D_{\Omega \backslash R}^{-1}J^{+})\left(\xi \right)\cdot \nu =(-\eta^{+}\nabla u^{+})\left(\xi \right)\cdot \nu \neq (D_{R}^{-1}J^{-})\left(\xi \right)\cdot \nu =(-\eta^{-}\nabla u^{-})\left(\xi \right)\cdot \nu . \end{array} \end{array}$$ (40)

Eqs (39) and (40) show the fundamental differences between MREIT and DT-MREIT. Eq (39) implies that it is nearly impossible to have any distinguishable contrast along an anomaly boundary if current flow is orthogonal to its tangent vector. In addition, Park *et al*. [15] show that it is possible to determine the conductivity uniquely if the conductivity value is known at the boundary. To avoid the ambiguity caused by (39), most MREIT algorithms use two independent currents to reconstruct unique isotropic conductivity distributions. On the other hand, (40) implies that a distinguishable contrast at tissue edges can obtained using measured diffusion tensors **D**, if diffusion values are different in the two regions. These assumptions are physically distinct, because diffusion coefficients are principally determined by the medium viscosity. Hence, incorporation of **D** allows the possibility of using only one current injection to reconstruct position dependent conductivity scale factors. However, from (.27) if ∇ln(*η*) is parallel to (**D**^−1^ **J**) vectors it is not possible to obtain any distinguishable boundary information using DT-MREIT.

**Fig 9:**
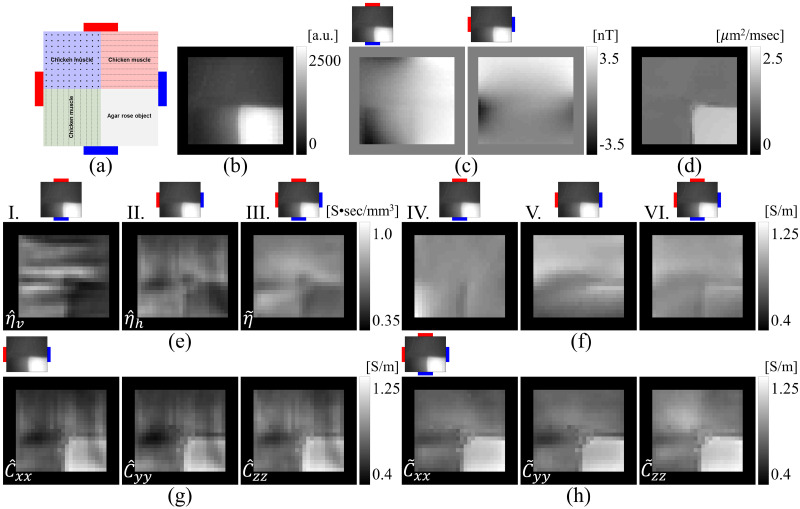
Example illustrating distinct MREIT and DT-MREIT properties. (a) Phantom design. (b)-(c) MR and corresponding $B_{z}^{m,E}\;E=1,2$ images. (d) Mean diffusivity map showing muscle directions. (e) Scale factor images reconstructed using dual-loop method for (I) horizontal, (II) vertical injection only. Part III shows the scale factor image obtained using two-current DT-MREIT method. (f) IV-V show equivalent isotropic conductivities from one current injection, and VI is isotropic conductivity found using two-current injections and the *J*-substitution algorithm [4]. Parts (g)-(h) show diagonal components of the reconstructed conductivity tensor using one-(horizontal) and two-current injection methods respectively. Images cropped to 40 × 40.

Fig 9(f) shows an example of the condition described above, where some tissue boundaries are not visible in single current injection MREIT data. However, because of the discontinuity at the tissue interface, DT-MREIT can distinguish the tissue boundary even with the single current injection shown in Fig 9(e). The interested reader may find details of this imaging experiment in S2 File.

### Phantom results

Single-current *L*^2^ differences were around 10% different from two-current DT-MREIT reconstructions. We found that the lowest longitudinal conductivities in the muscle samples were measured in the ‘top’ sample, where the tissue was oriented along the *z*-direction. This could have been related to the particular sample or limitations in the diffusion data. Overall, the anisotropic ratios of the ‘left’ (*x*-oriented) and ‘right’ (*y*-oriented) muscle samples measured in the diffusion tensor images were lower than expected (around 1.2). The top (*z*-oriented) sample had the lowest anisotropic ratio of 1.05, lower than that reconstructed for agar or potato samples in the same phantom. It is possible that anisotropic properties were not sampled adequately at 0.625 millimeter resolution. It may also be the case that there was little anisotropic property remaining in the muscle tissue samples.

### Assumption of boundary scaling factor values

Implementation of the dual-loop method requires an assumption of conductivity at the object boundary. We therefore specified the boundary scale factor as $\eta_{\partial \Omega }=(\frac{C_{e}}{d_{e}})=0.50\;\text{S}\cdot {\text{sec/mm}}^{3}$ based on the ratio of the known conductivity of the agar gel background (1 S/m) and the apparent diffusion tensor map measured in the agar gel background region. However, for the human data we assigned the scale factor value on the boundary to be *η*_∂Ω_ = 0.40 *S* ⋅ *sec*/*mm*^3^ [33]. This may have been responsible for some of the *L*^2^ differences observed in the phantom and human experimental results.

### Study limitations and future work

As noted in Lee *et al*. [34] and Sajib *et al*. [35], noise propagation along equipotential lines limits the performance of the dual-loop method. The degree of noise propagation depends on the condition number of the stiff matrix in Eq (9) [34, 35]. However, application of a total variation denoising technique, as in [35], to input data sets before performing dual-loop calculations may improve the condition number and reconstruction performance, but may not greatly reduce streaking artifacts, or reconstruction fidelity because of oversmoothing. It is also possible to reconstruct the scale factor using a single-loop method. However, one-loop reconstructions are strongly influenced by local changes of current flow, noise and reconstruction path [34].

In these studies, ANN methods were able to reconstruct the scale factors shown in Fig 5(c) and 5(d) for phantom data; or reconstruct human data, as in the *η* images of Fig 7 with reasonable accuracy using 1000 training data sets (S3 File), by employing the prior information provided by dual-loop estimations. While the agreement between standard two-current and single-current dual-loop reconstructions in these preliminary tests was encouraging, further improvements in machine learning algorithms may produce better overall performance. For example, one of the limitations in this study was that we used a general regression neural network (GRNN) to find the regressor function *f* in Eq (10). Because the centers and the weights in the pattern and summation unit of a GRNN network are determined from subject-specific training data sets, it cannot be generally extended to predict any artifact-free scale-factor image. To avoid this, in future studies we plan to use more diverse training data in conjunction with a deep learning model that is expected to improve reconstruction performance by taking into account local image features.

In future work, we intend to test these techniques *in vivo* in human heads for other tES electrode montages. In human tES studies it is rare to use in-plane (transverse) electrode montages, and electrode locations are instead chosen close to a presumed cortical target [11]. As noted previously, the difference between projected current density and true current density estimates depends on the out of plane current component *J*_*z*_ [15, 16]. For transverse currents, nearly all current density information is encoded in *B*_*z*_ data, and *J*_*z*_ data is negligible. The quality of projected current density data is key to both dual-loop (9) and DT-MREIT reconstructions (27). Therefore, we expect that the single-current data reconstructions used here will become less accurate as *J*_*z*_ increases. Deep learning methods are also anticipated to be of potential benefit for these more general situations. Training data for deep learning could be generated using partial dual-loop-reconstructed data from off-plane montages or computed predictions of these data with arbitrary electrode locations.

## Conclusion

We tested a novel approach to reconstructing apparent conductivity tensor and corresponding electric field distributions in magnetic resonance electrical impedance tomography applications using an artificial neural network, with training data informed by mimetic algorithm data. Use of these methods may be useful in obtaining conductivity, current density and electric field measurements where it is only possible to measure magnetic flux density data resulting from a single current administration. We believe that the method will be useful for monitoring electromagnetic field distributions and optimizing stimulation protocols used in human tES studies [54, 55].

## Supporting information

S1 File Stray magnetic field correction. (PDF)

S2 File Description of the block phantom. (PDF)

S3 FileEffect of data sample size M on reconstruction performance.(PDF)

S4 File Dataset information. (PDF)
