# Supplementary material for: Magnetic-resonance-based measurement of electromagnetic fields and conductivity in vivo using single current administration—A machine learning approach
Source: PLoS One. 2021 Jul 22;16(7):e0254690. doi: 10.1371/journal.pone.0254690 (PMC8297925; doi:10.1371/journal.pone.0254690)
Supplement: S1 File — (PDF) [file pone.0254690.s001.pdf]

Magnetic-resonance-based measurement of electromagnetic fields and conductivity *in vivo* using single current administration - a machine learning approach

S. Z. K. Sajib, M. Chauhan, O. I. Kwon, R. Sadleir*
* rjsadleir@asu.edu

S1 Stray magnetic field correction

In MREIT, the induced B_z^m due to external current injection is collected using an MRI scanner. The acquired MREIT signal inside the MRI scanner can be expressed as,

$$\tilde{B}_z^m(\mathbf{r}) = \frac{\mu_0}{4\pi}\int_\Omega \frac{(y-y^{'})J_x(\mathbf{r}^{'})-(x-x^{'})J_y(\mathbf{r}^{'})}{|\mathbf{r}-\mathbf{r}^{'}|^3}d\mathbf{r}^{'} + B_{z,\mathcal{L}}(\mathbf{r}) \tag{S1}$$

where μ_0 is the magnetic permeability of free space, $\mathbf{r}$ is the position vector in three dimensional space, and $\mathbf{J} = [J_x, J_y, J_z]$ is the current density formed inside the domain Ω. The first term of equation (S1) describes the magnetic flux density produced by the external current injection inside the domain, and depends on the boundary conditions, domain shape and conductivity contrast distribution. The second term describes the magnetic flux density recorded inside the domain caused by current flow in the lead wires $\mathcal{L}$, leading to the domain. This contribution can be effectively minimized if the wires are aligned along the main magnetic field direction (z-direction) as much as possible [20]. However, for practical TES setups, it is almost impossible to align wires along the z-direction. Hence, it is important to take wire-related stray magnetic fields into account [50,51].

Stray field corrections were performed by detecting wire paths during experiments, and computing and removing wire contributions. The wire model was created from high resolution T_1-weighted images gathered as part of regular imaging protocols. Wires were encapsulated with silicone tubing (Tygon 3350 Sanitary Silicone Tubing, Saint Gobain, Paris, France) to enable their detection in structural sequences. A three-dimensional tube mask was first created from these high resolution T_1-weighted images. The segmentation was performed in ScanIP (Synopsys Inc., Mountain View, CA, USA) software. Segmented tube masks were then exported to MATLAB and wire trajectories were determined from mask centroids Fig. (4a). Stray magnetic field contributions ($\mathbf{B}_\mathcal{L} = [B_{x,\mathcal{L}}, B_{y,\mathcal{L}}, B_{z,\mathcal{L}}]$) were computed as

$$\mathbf{B}_\mathcal{L}(\mathbf{r}) = \frac{I\mu_0}{4\pi}\int_\mathcal{L} \hat{\mathbf{a}}(\mathbf{r}^{'}) \times \frac{\mathbf{r}-\mathbf{r}^{'}}{|\mathbf{r}-\mathbf{r}^{'}|}dl^{'}$$

where I is amplitude of current and $\hat{\mathbf{a}}$ is the unit vector in the direction of the current flow at $\mathbf{r}^{'} \in \mathcal{L}$. Computed $B_{z,\mathcal{L}}$ fields were subtracted from acquired $\tilde{B}_z^m$ data to obtain corrected magnetic fields B_z^m.

Results were compared against numerical simulations. Simulated data were obtained by solving the Laplace equation within the homogeneous head model to obtain current density distributions using the COMSOL-MATLAB interface (COMSOL Inc., Burlington, MA, USA). The conductivity of the domain Ω was set to 1 S/m. Simulated B_z data were then computed from the calculated current density distribution using the Biot-Savart law.

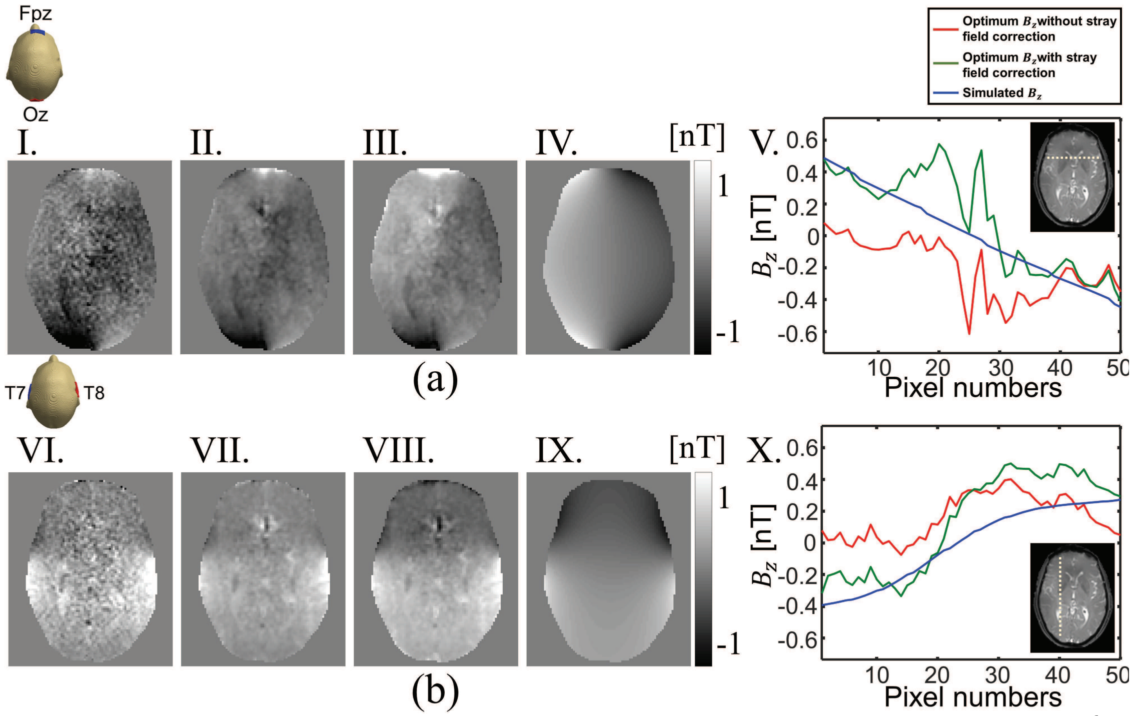

Fig S1. Stray field correction for human experimental data. Images (a-I) and (b-VI) show 5th echo of measured multi-gradient echo phase images representing the z-component of magnetic flux density induced due to 1.5 mA current injection through the Fpz-Oz and T8-T7 electrode pairs respectively. Parts (a-II) and (b-VII) show optimum combinations of $\tilde{B}_z^m$ images, without stray field correction. Images (a-III) and (b-VIII) show stray-magnetic-field-corrected optimized B_z^m images. Parts (a-IV) and (b-IX) show simulated domain-induced magnetic flux densities obtained using a numerical model. Profile plots (a-V) and (b-X) compare effectiveness of stray field corrections for Fpz-Oz and T8-T7 electrode montages respectively. All B_z images are masked to the brain region and cropped to 85×100.
