# Supplementary material for: Magnetic-resonance-based measurement of electromagnetic fields and conductivity in vivo using single current administration—A machine learning approach
Source: PLoS One. 2021 Jul 22;16(7):e0254690. doi: 10.1371/journal.pone.0254690 (PMC8297925; doi:10.1371/journal.pone.0254690)
Supplement: S2 File — (PDF) [file pone.0254690.s002.pdf]

Magnetic-resonance-based measurement of electromagnetic fields and conductivity *in vivo* using single current administration - a machine learning approach

S. Z. K. Sajib, M. Chauhan, O. I. Kwon, R. Sadleir*
* rjsadleir@asu.edu

S2 Description of the block phantom

Three pieces of chicken muscle, oriented along x, y, and z-directions respectively, were placed, along with a piece of isotropic agar (∼1 S/m) into a sample chamber. The sample chamber was an acrylic rectangular shaped vessel with dimensions $33 \times 35 \times 28$ mm^3. All measurements were acquired using a 7-T Bruker (Bruker Biospin MRI, Billerica, MA, USA) scanner equipped with a single channel RF volume coil. Current amplitudes of 10 mA were delivered via opposing pairs of carbon electrodes (10 mm × 10 mm). Sets of $B_z^{m,\mathcal{E}}$, $\mathcal{E} = 1, 2$ data for the current pairs were collected using a spin-echo MR pulse sequence. Imaging parameters used for B_z data collection were, TR/TE = 1000/20 ms, field of view, FOV = 64 mm × 64 mm, image matrix size 64×64, slice thickness 4 mm (no slice gap) and number of slices NS = 7. Diffusion weighted images (DWI) of these seven slices were also collected, using a single-shot spin-echo echo planar imaging sequence, using the same matrix size, FOV and slice thickness as in MREIT experiments. Six different gradient directions were used, with b-values of 1000 sec/mm^2. Parameters used for DWI scans were TR/TE = 2300/27 ms.
