# Supplementary material for: Magnetic-resonance-based measurement of electromagnetic fields and conductivity in vivo using single current administration—A machine learning approach
Source: PLoS One. 2021 Jul 22;16(7):e0254690. doi: 10.1371/journal.pone.0254690 (PMC8297925; doi:10.1371/journal.pone.0254690)
Supplement: S3 File — (PDF) [file pone.0254690.s003.pdf]

Magnetic-resonance-based measurement of electromagnetic fields and conductivity *in vivo* using single current administration - a machine learning approach

S. Z. K. Sajib, M. Chauhan, O. I. Kwon, R. Sadleir*
* rjsadleir@asu.edu

S3 Effect of data sample size $\mathcal{M}$ on reconstruction performance

We also investigated reconstruction performance with respect to training data sample size $\mathcal{M}$ by measuring the relative L^2 error RE (34) and mean structural similarity index $MSSIM$ (36) at sample data sizes of $\mathcal{M} = 250, 500, 750$. For each training data set the network was optimized for α. For vertical projection phantom data ($\mathcal{E} = 1$) optimum spread constant values were found to be $0.94, 0.92$ and 0.89 for sample sizes $\mathcal{M} = 250, 500, 750$, respectively, whereas for $\mathcal{E} = 2$ (horizontal) $\hat{\alpha}$ values were found to be $0.81, 0.78$ and 0.76. For the human subject data and the Fpz-Oz projection ($\mathcal{E} = 1$) optimum spread constant values were $2.09, 2.07$ and 1.92, and for the T7-T8 projection ($\mathcal{E} = 2$) $\hat{\alpha}$ values were $1.31, 1.16, 1.09$, for data sample sizes of $250, 500$ and 750 respectively.

As anticipated, the RE values decreased and $MSSIM$ values increased as the sample size (or the number of neurons in the pattern layer) increased from 250 to 750 [36] and the best results were obtained with a finite data-sample and $\mathcal{M} = 1000$ (Fig. S3).

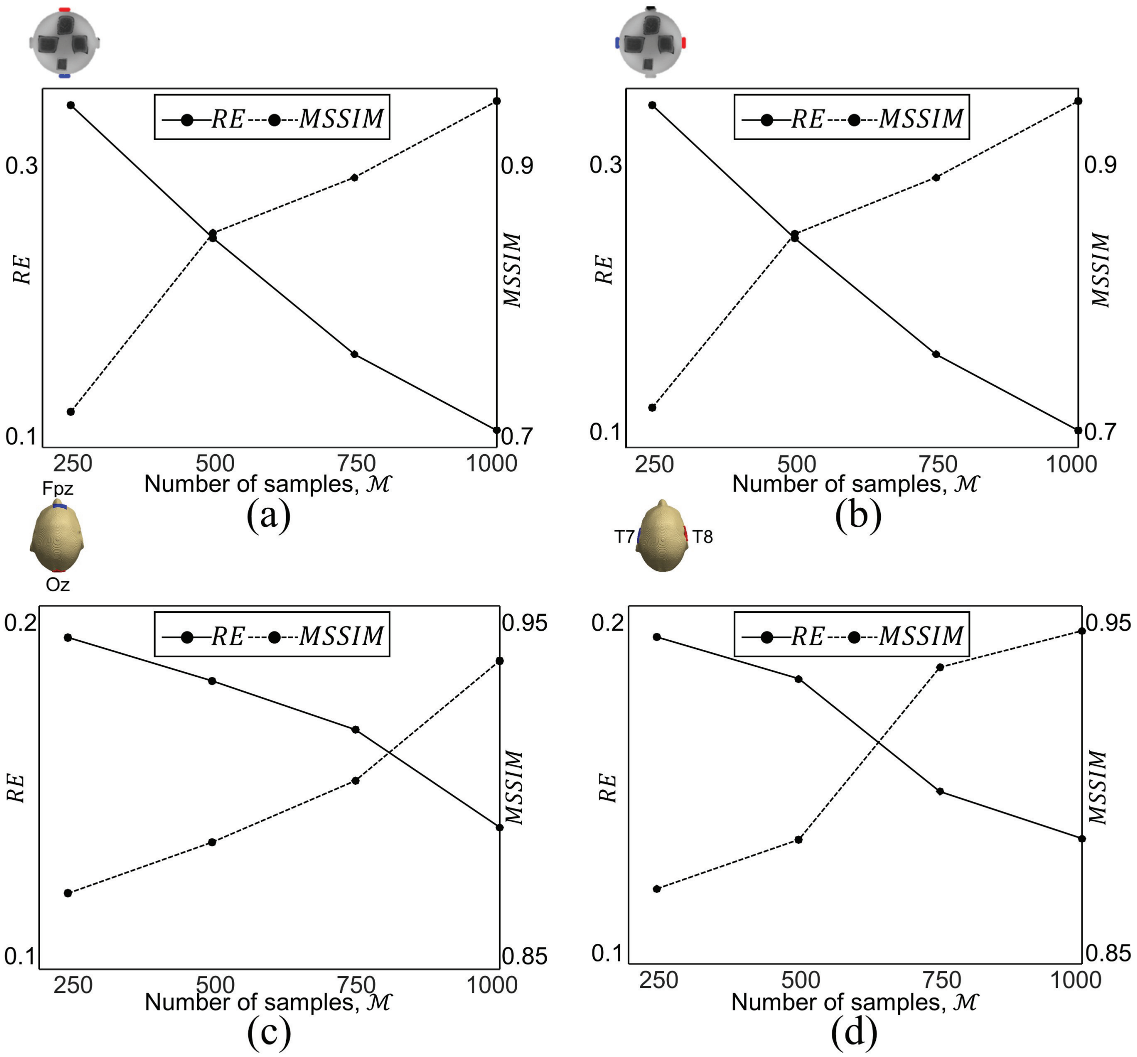

Fig S3. Dependence of predicted output of GRNN on sample size $\mathcal{M}$. All networks were optimized using the holdout method at data sample sizes $\mathcal{M} = 250, 500, 750$. Reconstructed RE and $MSSIM$ for vertical ($\mathcal{E} = 1$) and horizontal ($\mathcal{E} = 2$) current injection phantom data are shown in parts (a) and (b), respectively. Phantom data RE and $MSSIM$ values were calculated from the total image volume. Parts (c)-(d) show reconstruction performance plots for the Fpz-Oz and T7-T8 electrode montages in human subject data, respectively.
