# Supplementary material for: Magnetic-resonance-based measurement of electromagnetic fields and conductivity in vivo using single current administration—A machine learning approach
Source: PLoS One. 2021 Jul 22;16(7):e0254690. doi: 10.1371/journal.pone.0254690 (PMC8297925; doi:10.1371/journal.pone.0254690)
Supplement: S4 File — (PDF) [file pone.0254690.s004.pdf]

Magnetic-resonance-based measurement of electromagnetic fields and conductivity *in vivo* using single current administration - a machine learning approach

S. Z. K. Sajib, M. Chauhan, O. I. Kwon, R. Sadleir*
* rjsadleir@asu.edu

S4 Dataset Information

Raw data required to reproduce computations in this paper for the phantom may be found at the ASU Research Database Repository dataverse.asu.edu via the following `dataverse.asu.edu/dataset`.

Human Subject data may be accessed at the National Institute of Mental Health Data Archive at `nda.nih.gov`.

Format and access information for each dataset contained in Table S4.

Table S4. Description of data files.

Dataset	Structural MRI		MREIT experiment		Diffusion experiment	
Phantom [a]	T1	—	MR	$128 \times 128 \times 5$	DWI [b]	$128 \times 128 \times 5 \times 7$
			Bz	$128 \times 128 \times 5$	gradient [c]	7×3
Human [d]	T1	$224 \times 224 \times 224$	MR	$128 \times 128 \times 3 \times 10 \times 2$	DWI	$128 \times 128 \times 3 \times 16$
			Bz	$128 \times 128 \times 3 \times 10 \times 2$	gradient	16×3

[a] https://dataverse.asu.edu/dataset.xhtml?persistentId=doi:10.48349/ASU/2T05EL
[b] diffusion weighted image
[c] diffusion sensitized gradient
[d] nda.nih.gov. Data Collection ID C2750.
